# Supplementary material for: HDAC11 inhibition triggers bimodal thermogenic pathways to circumvent adipocyte catecholamine resistance
Source: bioRxiv. 2023 Mar 30:2023.03.29.534830. Preprint. [Version 1] doi: 10.1101/2023.03.29.534830 (PMC10081236; doi:10.1101/2023.03.29.534830)
Supplement: Supplement 1 — Supplemental Figure 1. Validation of FT895 selectivity for HDAC11. Supplemental Figure 2. Validation of UCP1 antibody specificity. [file media-1.pdf]

Supplemental Figure 1

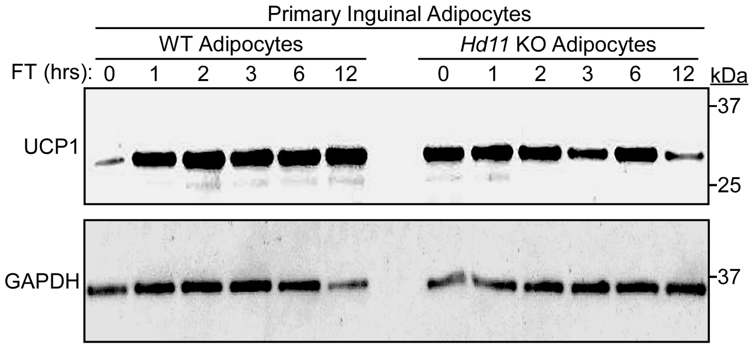

Supplemental Figure 1. Validation of FT895 selectivity for HDAC11. Primary adipocytes were isolated from inguinal white adipose tissue from WT and *Hdac11* global knockout (*Hd11* KO) mice and were exposed, in culture, to FT895 for the indicated times prior to homogenization and immunoblotting to assess UCP1 expression; GAPDH served as a loading control.

Supplemental Figure 2

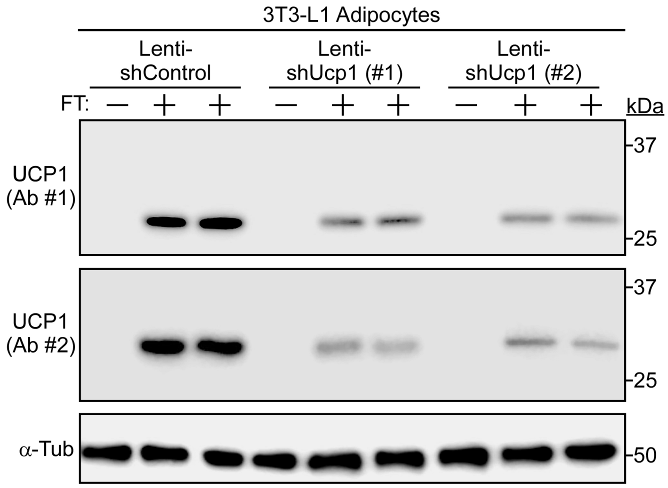

Supplemental Figure 2. Validation of UCP1 antibody specificity. 3T3-L1 adipocytes were infected with lentivirus encoding a control short-hairpin RNA (Lenti-shControl) or viruses encoding two independent shRNAs to target *Ucp1* mRNA (shUcp1). Cells were homogenized 24-hours after infection and immunoblot analysis was performed to assess UCP1 protein expression; α-tubulin (α-Tub) served as a loading control.
