# Supplementary material for: HDAC11 inhibition triggers bimodal thermogenic pathways to circumvent adipocyte catecholamine resistance
Source: bioRxiv. 2023 Mar 30:2023.03.29.534830. Preprint. [Version 1] doi: 10.1101/2023.03.29.534830 (PMC10081236; doi:10.1101/2023.03.29.534830)
Supplement: Supplement 2 — Supplemental Table 1. Primer sequences for genotyping, qRT-PCR and short hairpin RNA. Supplemental Table 2. Chemicals and reagents. Supplemental Table 3. Patient information for human VAT samples. Supplemental Table 4. Patient information for human SC adipocyte isolation. Supplemental Table 5. Antibodies. [file media-2.pdf]

Supplemental Table 1. Primer sequences for genotyping and qRT-PCR

Primer Name	Application	Species	Primer Sequence
Ucp1 F	RT-qPCR	Human	5’-AGTTCCTCACCGCAGGGAAAGA-3’
Ucp1 R	RT-qPCR	Human	5’-GTAGCGAGGTTTGATTCCGTGG-3’
Ucp1 F	RT-qPCR	Mouse	5’-CCGAAACTGTACAGCGGTCT-3’
Ucp1 R	RT-qPCR	Mouse	5’-CCGAGAGAGGCAGGTGTTTC-3’
18S F	RT-qPCR	Human, Mouse	5’-GCCGCTAGAGGTGAAATTCTTA-3’
18S R	RT-qPCR	Human, Mouse	5’-CTTTCGCTCTGGTCCGTCTT-3’
Hdac11 flox F	DNA PCR	Mouse	5’-GTGCAGGCCTTGGGCCTTGGCA-3’
Hdac11 flox R	DNA PCR	Mouse	5’-CTGAGGAGGTAGTATGGATAG-3’

Supplemental Table 2. Chemicals and reagents

Item	Vendor	Catalog #
Dulbecco's Modification of Eagle's Medium (DMEM)	Corning	10-013-CV
(Minimum Essential Medium α (MEMα)	Corning	10-022-CV
Fetal Bovine Serum, HyClone Characterized FBS, US Origin	GE Healthcare	SH30071.02
Newborn Calf Serum, heat inactivated, New Zealand origin	Thermo Scientific	26010074
Penicillin-Streptomycin-L-Glutamine, 100X	Corning	30-009-CI
Insulin-Transferrin-Selenium, 100X	Thermo Gibco	41400045
Dexamethasone	Sigma Aldrich	D4902
3-Isobutyl-1-methylxanthine, IBMX	Sigma Aldrich	I5879
Rosiglitazone	Cayman Chemicals	71740
Collagenase II	Worthington	LS004176
Cell-permeable Cre recombinase, TAT-Cre (Tat-NLS-Cre, HTNC, HTNCre)	Excellgen	EG-1001
Halt™ Protease and Phosphatase Inhibitor Cocktail	Thermo Scientific	78440
Pierce™ BCA Protein Assay Kit	Thermo Scientific	PI23227
4-15% Criterion TGX Precast Midi Protein Gel, 26 well,	BIO-RAD	5671085
4-15% Criterion TGX Precast Midi Protein Gel, 18 well	BIO-RAD	5671084
Nitrocellulose membrane 0.45 µm	BIO-RAD	1620115
Precision Plus Protein Dual Color Standards	BIO-RAD	1610394
Bovine Serum Albumin Fraction V for immunoblotting	Akron Biotech	AAJ64655
Bovine Serum Albumin, fatty acid free for immunofluorescence	Thermo Scientific	A8806
Stainless steel beads 1.6 mm	Next Advance	#SSB16
Alkynyl Myristic Acid (Alk-12)	Click Chemistry Tools	1164

Click-iT® Protein Reaction Buffer Kit	Thermo Scientific	C10276
Biotin Azide (PEG4 carboxamide-6-Azidohexanyl Biotin)	Thermo Scientific	B10184
Pierce™ Streptavidin Magnetic Beads	Thermo Scientific	88816
FT895; FT	MedChemExpress	2225728-57-2
CL-316,243	Sigma Aldrich	C5976
Isoproterenol; ISO	Calbiochem	420355
Forskolin; FSK	Sigma Aldrich	F6886
H89	Tocris Bioscience	2910
DMSO	Sigma Aldrich	D8418
N,N-Dimethylacetamide (DMA)	Sigma Aldrich	D137510
Tween-80	Sigma Aldrich	P1754
4,4-Difluoro-1,3,5,7,8-Pentamethyl-4-Bora-3a,4a-Diaza-s-Indacene; BODIPY	Thermo Scientific	D3922
4',6-diamidino-2-phenylindole; DAPI	Thermo Scientific	D1306
D-Biotin	Sigma Aldrich	B4639
D-Pantothenic acid hemicalcium salt	Sigma Aldrich	P5155
3,3′,5-Triiodo-L-thyronine sodium salt; T3	Sigma Aldrich	T6397
Transferrin, human	Sigma Aldrich	T8158
Insulin solution, human	Sigma Aldrich	I9278
Tween-20	Sigma Aldrich	P1754
Glass coverslips, 25 mm	Chemglass	CLS-1763-025
Microscope slides, Diamond White Glass, 25 x 75mm, Charged, 90° Ground Edges, White Frosted	Global Scientific	1358W
Nunc™ Lab-Tek™ II Chamber Slide™	Thermo Scientific	12-565-7
Formaldehyde,16 % methanol-free Ultra Pure	Thermo Scientific	18814-10
VECTASHIELD® HardSet™ Antifade Mounting Medium for immunofluorescence	Vector Labs	H-1400-10
Antigen Unmasking Solution, Citrate-Based	Vector Labs	H-3300-250
BLOXALL® Endogenous Blocking Solution, Peroxidase and Alkaline Phosphatase	Vector Labs	SP-6000-100
ECTASTAIN® Elite® ABC-HRP Kit, Peroxidase (Standard)	Vector Labs	PK-6100
Vector® NovaRED™ Substrate Kit, Peroxidase (HRP)	Vector Labs	SK-4800
Fisher Chemical™ Permount™ Mounting Medium for IHC	Thermo Scientific	SP15-100
Agarose	Life Science Products	A-1705
1kb DNA ladder	Promega	PR-G5711
Protein ladder, Precision Plus Protein™ Dual Color Standards	BIO-RAD	1610394
Non-fat milk powder, Kroger® Instant Non-Fat Dry Milk	Kroger®	0001111083297
PowerUp SYBR Green Master Mix	Thermo Scientific	4368577

TRIzol™ Reagent	Thermo Scientific, Ambion	15596026
Verso cDNA Synthesis Kit	Thermo Scientific	AB1453B
Polybrene	Sigma Aldrich	TR-1003
Polyethylenimine, Linear, PEI	Polysciences Inc.	23966-1

Supplemental Table 3. Patient information for human VAT samples

Age (yrs)	Sex	Ethnicity	BMI Consent	BMI Surgery
30	F	Hispanic	42.0	42.0085992
39	M	Non-Hispanic, Indian	36.9839009	36.9839009
22	F	White	42.0	41.8102305

Supplemental Table 4. Patient information for human SC adipocyte isolation

Age (yrs)	Sex	BMI
29	M	21.1

Supplemental Table 5. Antibodies

Antigen	Vendor	Host	Catalog #
UCP1 #1	Abcam	Rabbit	ab209483
UCP1 #2	Cell Signaling Technology	Rabbit	14670S
GAPDH	Thermo Scientific	Mouse	AM4300
Phospho-PKA substrates	Cell Signaling Technology	Rabbit	9624
α-Tubulin-HRP	Santa Cruz Biotechnology	Mouse	sc-23948
Gravin-α	Proteintech	Rabbit	25199-1-AP
FLAG-HRP	Sigma Aldrich	Mouse	F1804
β_3-AR	Abcam	Rabbit	ab94506
β_2-AR	Proteintech	Rabbit	13096-1-AP
HDAC11	From Dr. Edward Seto, George Washington University	Rabbit	
anti-Rabbit Alexa Fluor 594 for Immunofluorescence	Thermo Scientific	Goat	A-11012
Anti-Rabbit IgG-HRP	Southern Biotech	Goat	OB4050-05
Anti-Mouse IgG-HRP	Southern Biotech	Goat	OB1031-05
Anti-Rabbit IgG Antibody (H+L), Biotinylated for IHC	Vector Labs	Goat	BA-1000-1.5
