# Supplementary material for: HDAC11 inhibition triggers bimodal thermogenic pathways to circumvent adipocyte catecholamine resistance
Source: bioRxiv. 2023 Mar 30:2023.03.29.534830. Preprint. [Version 1] doi: 10.1101/2023.03.29.534830 (PMC10081236; doi:10.1101/2023.03.29.534830)
Supplement: Supplement 4 [file media-4.pptx]

## Slide 1
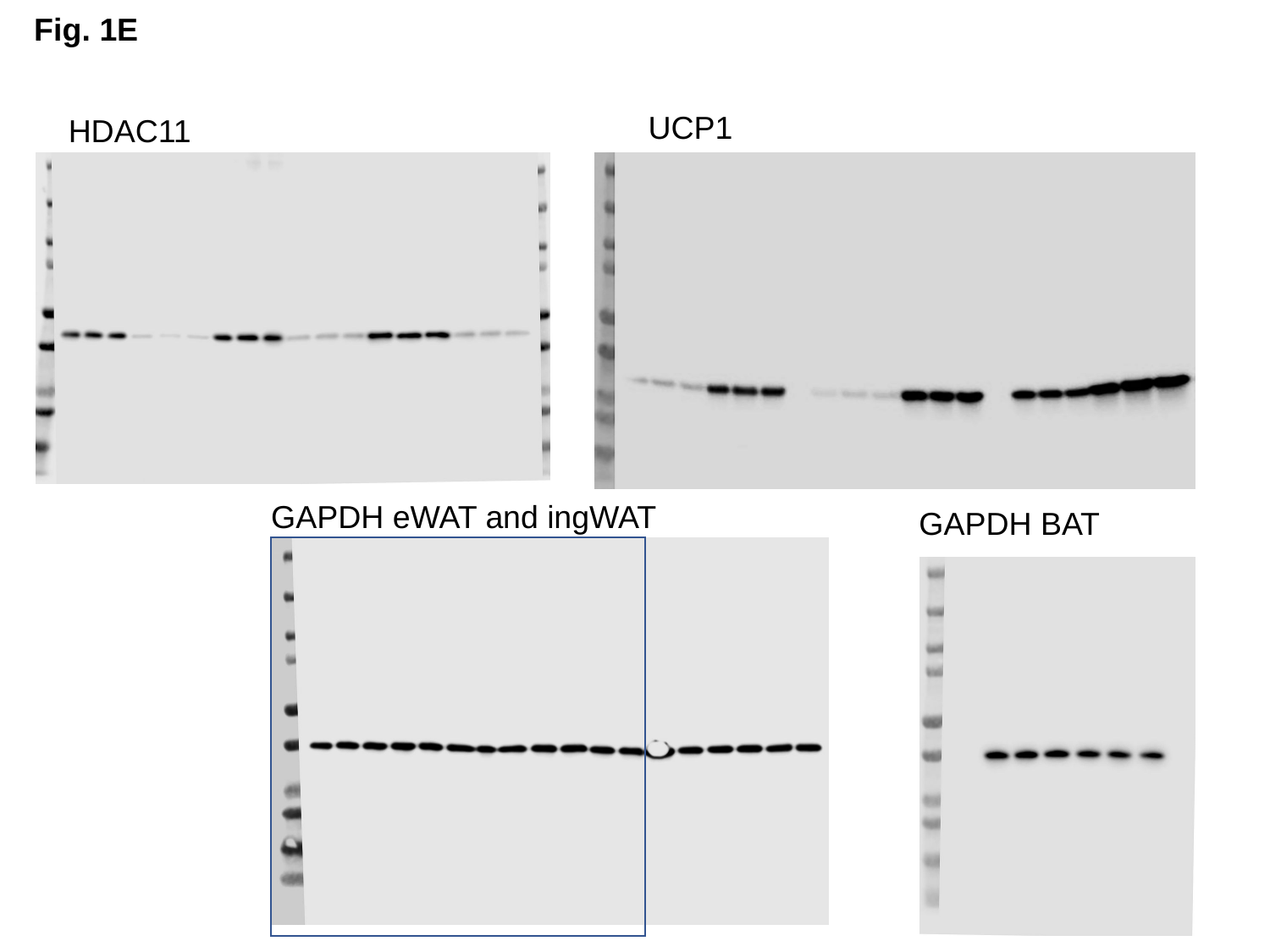

Fig. 1E
UCP1
HDAC11
GAPDH eWAT and ingWAT
GAPDH BAT

## Slide 2
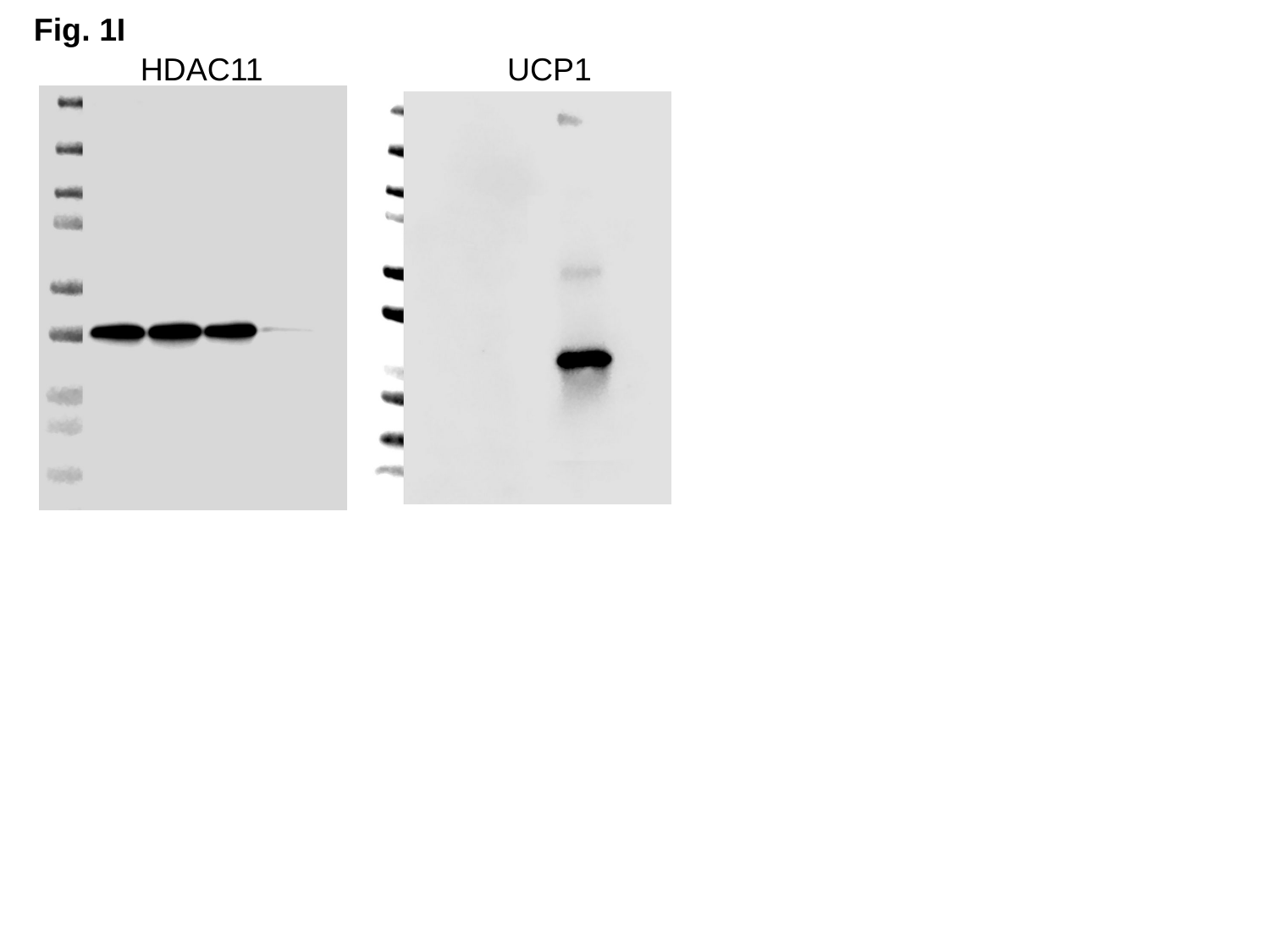

Fig. 1I
HDAC11
UCP1

## Slide 3
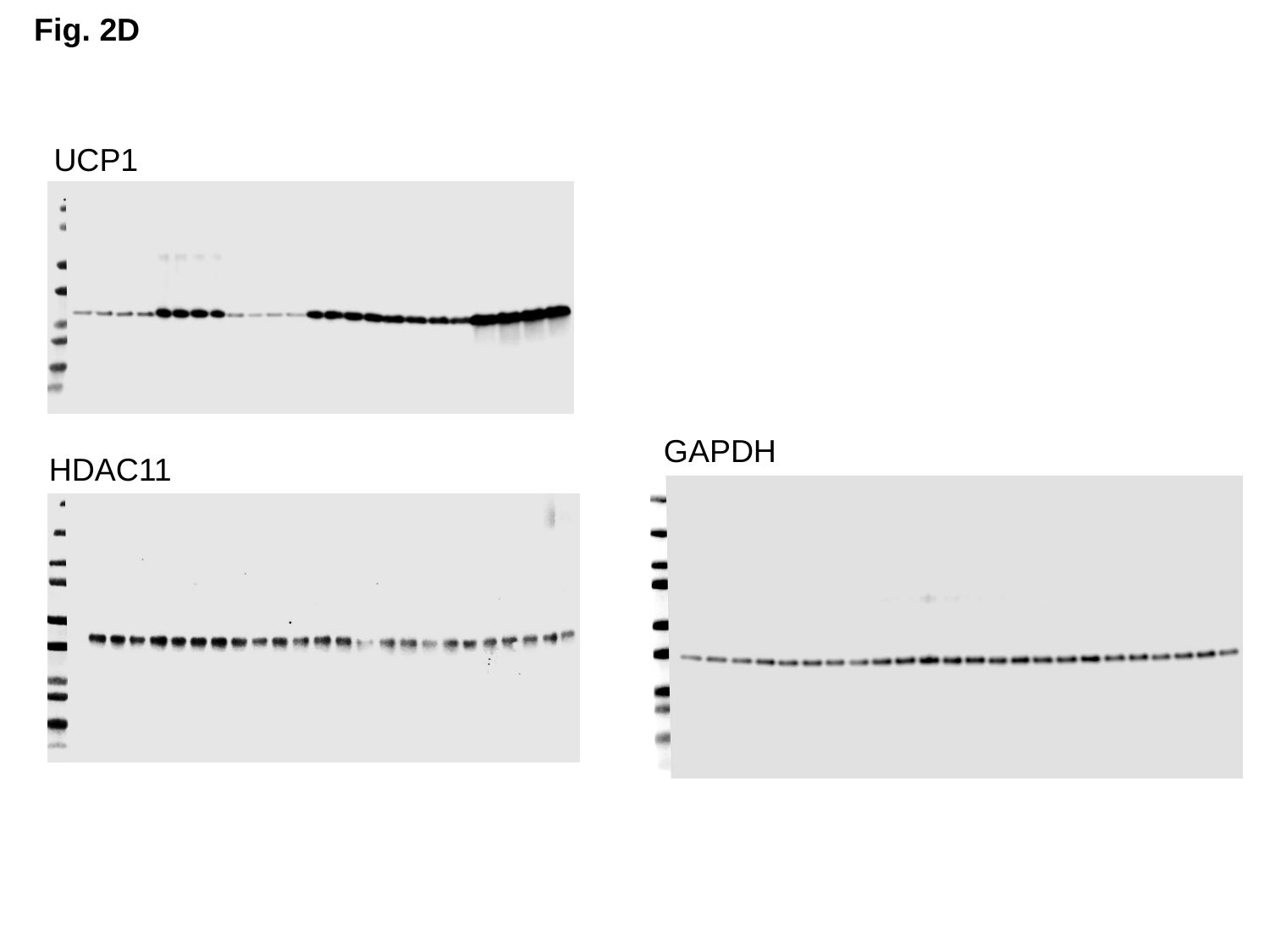

Fig. 2D
UCP1
GAPDH
HDAC11

## Slide 4
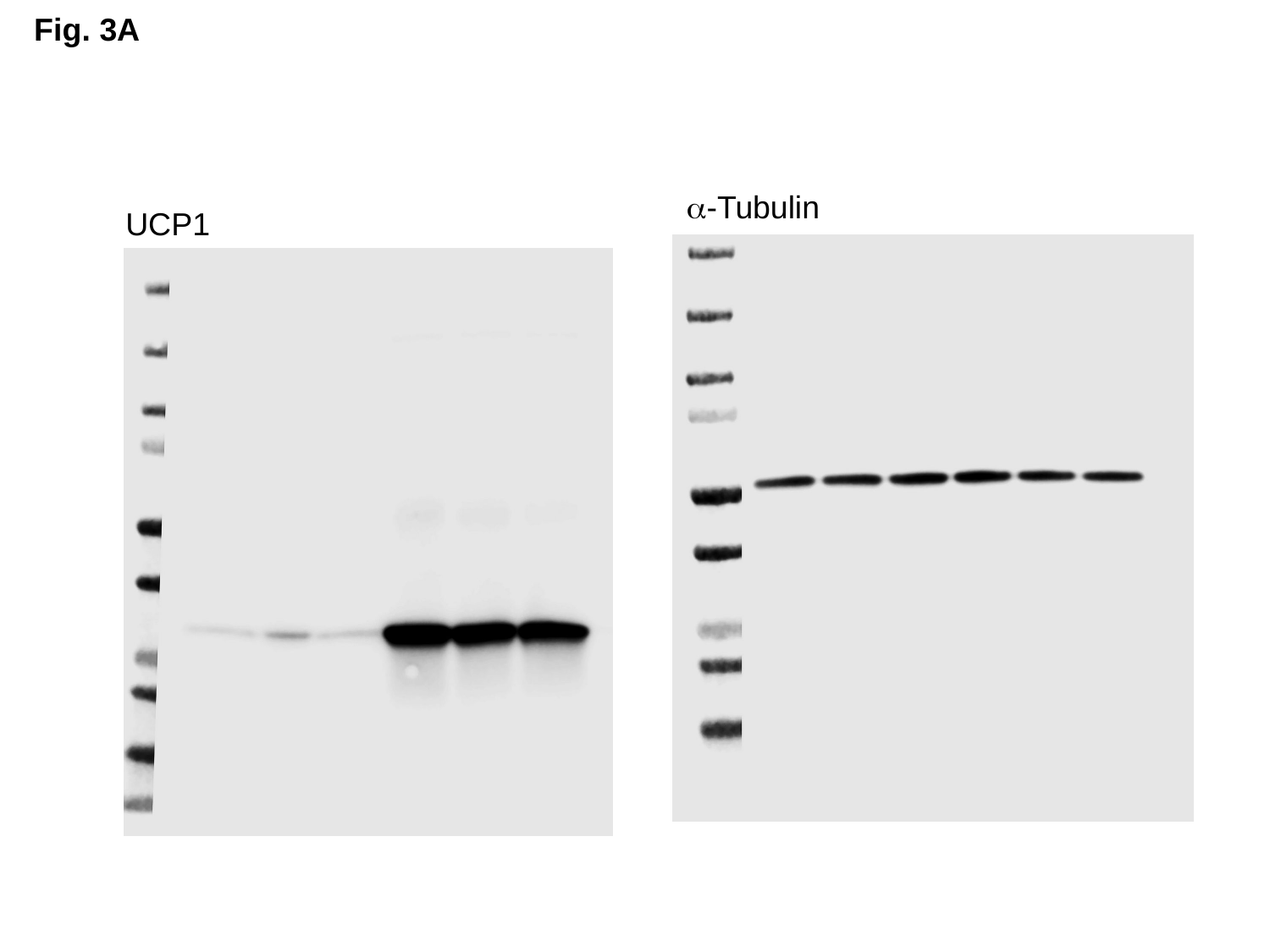

Fig. 3A
a-Tubulin
UCP1

## Slide 5
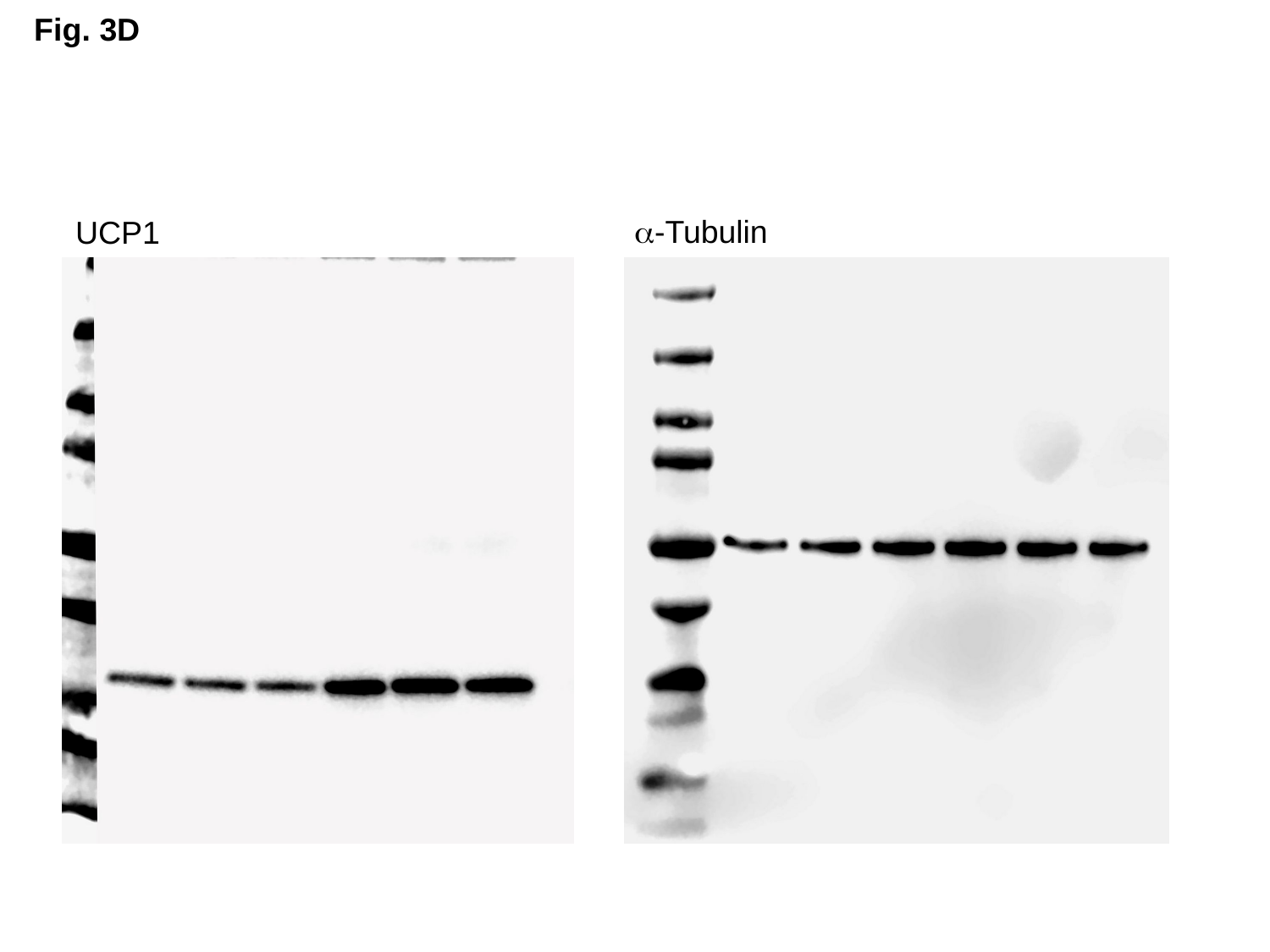

Fig. 3D
a-Tubulin
UCP1

## Slide 6
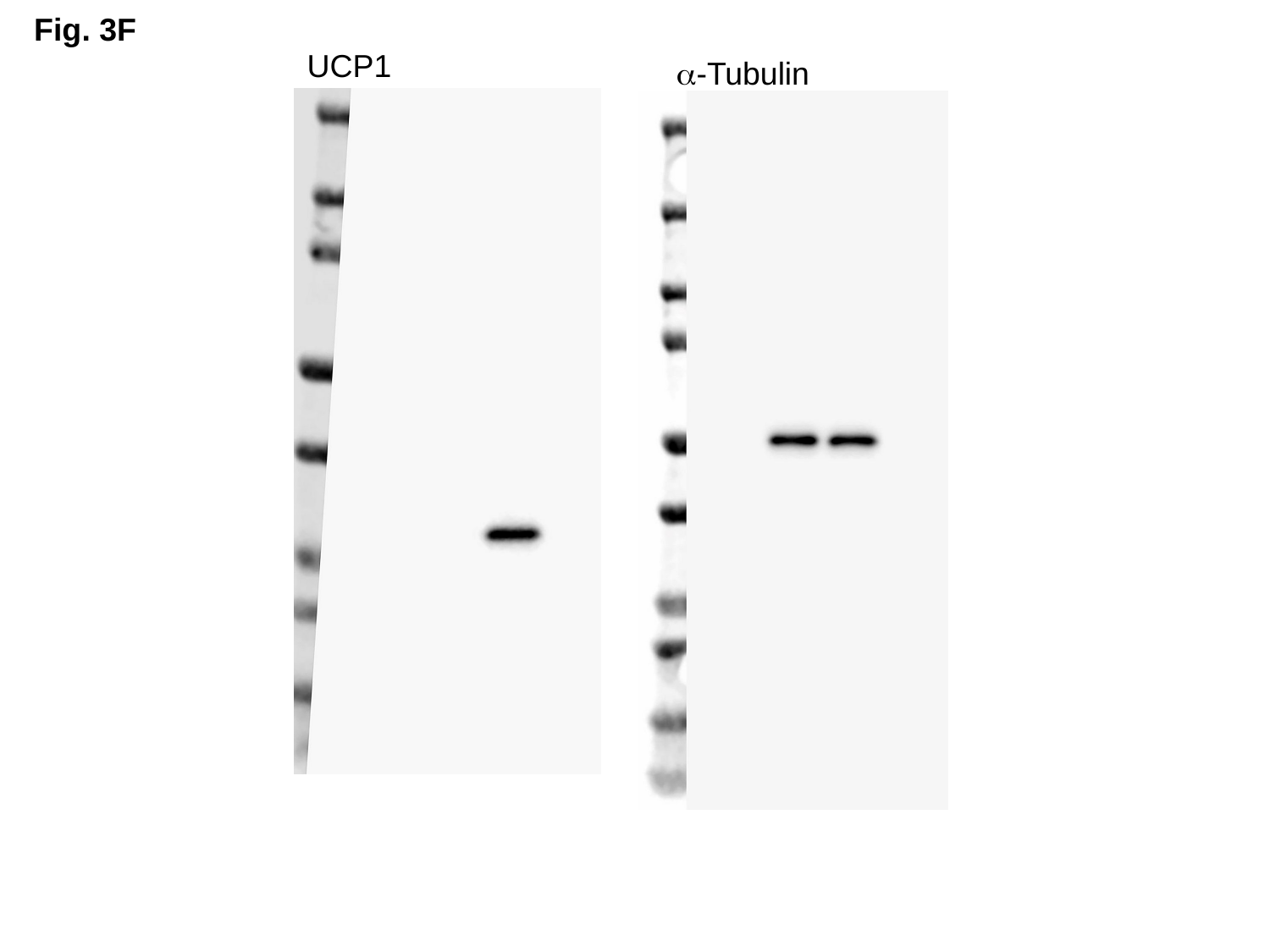

Fig. 3F
UCP1
a-Tubulin

## Slide 7
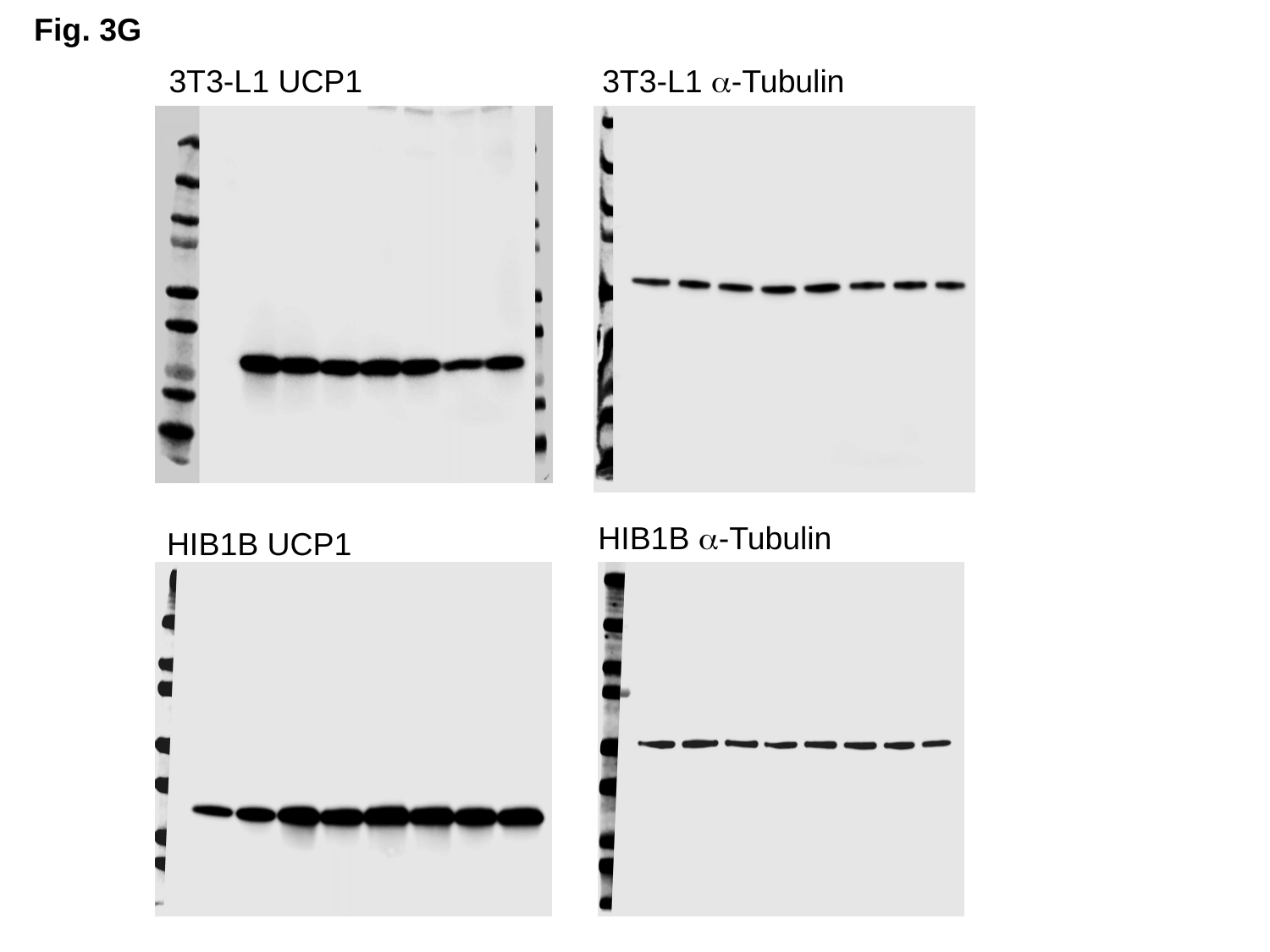

Fig. 3G
3T3-L1 UCP1
3T3-L1 a-Tubulin
HIB1B a-Tubulin
HIB1B UCP1

## Slide 8
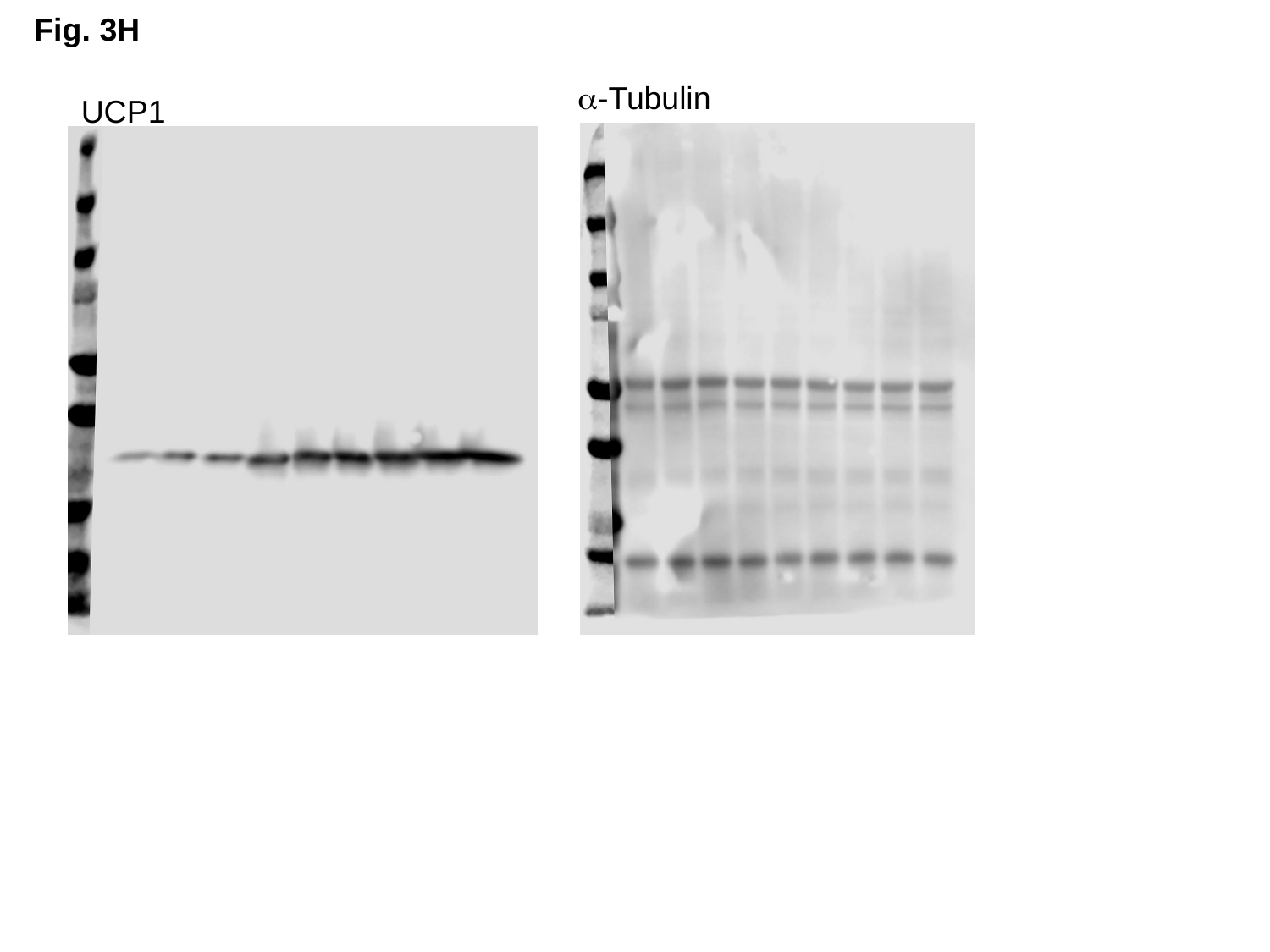

Fig. 3H
a-Tubulin
UCP1

## Slide 9
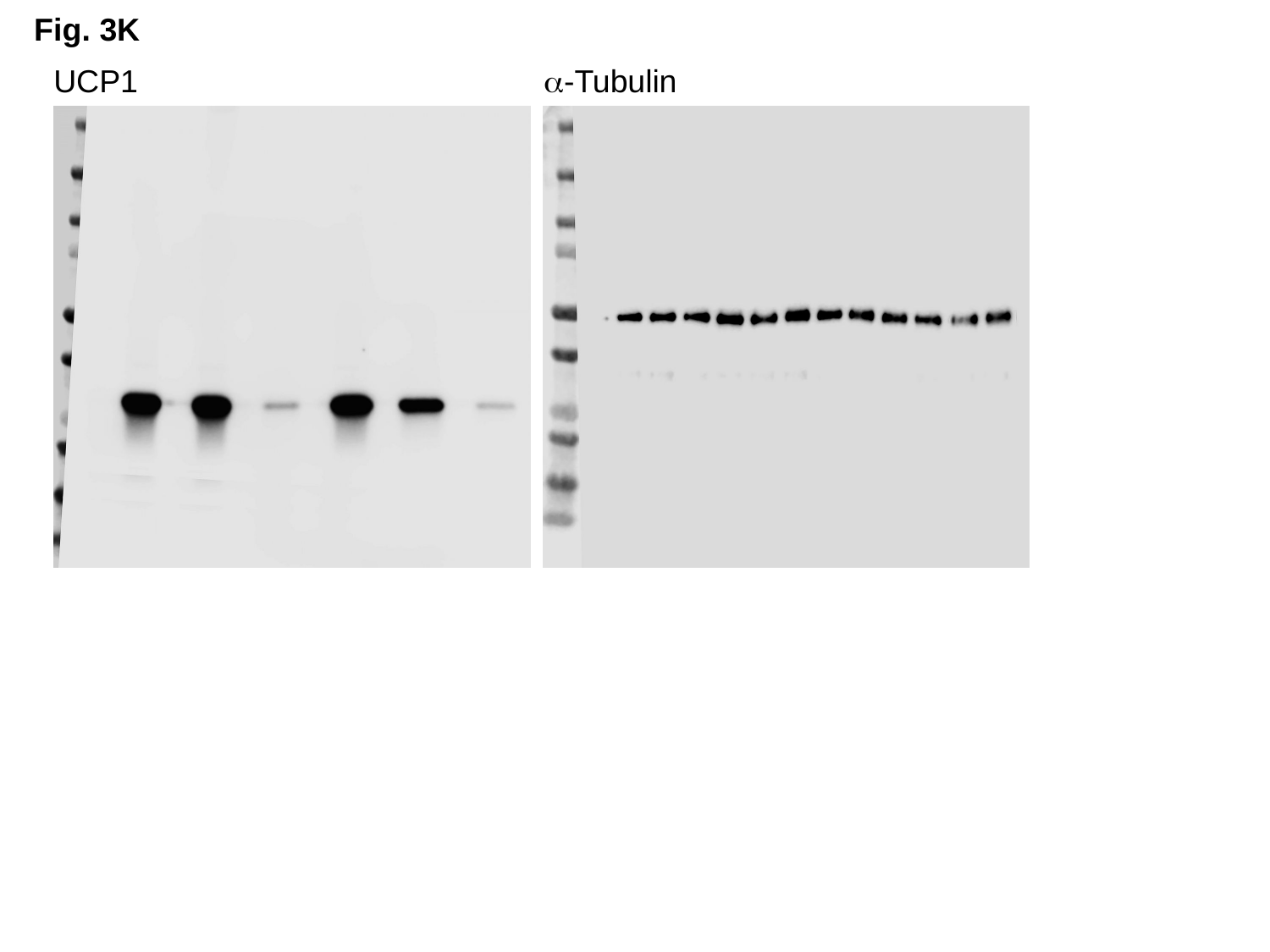

Fig. 3K
UCP1
a-Tubulin

## Slide 10
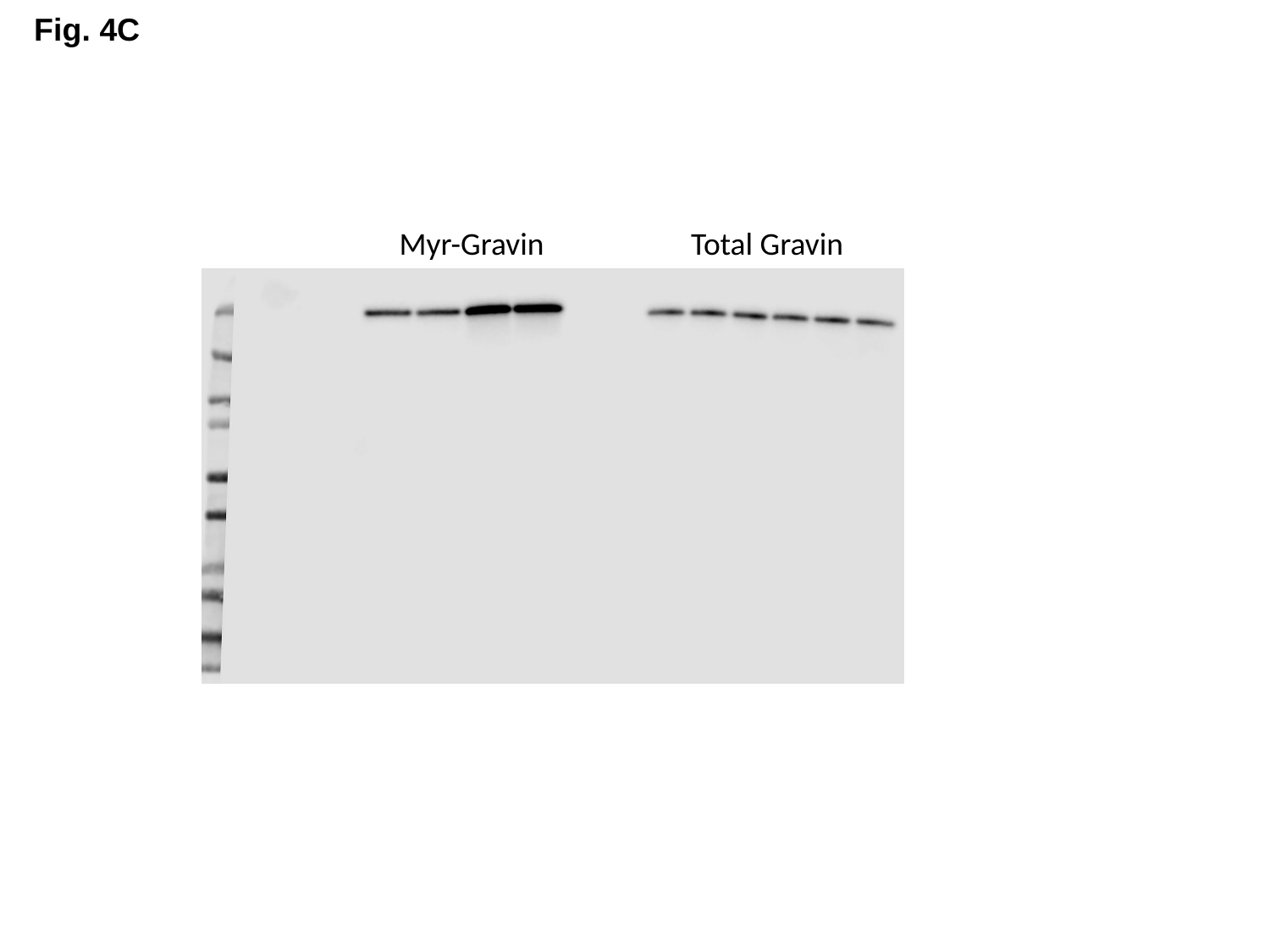

Fig. 4C
Myr-Gravin
Total Gravin

## Slide 11
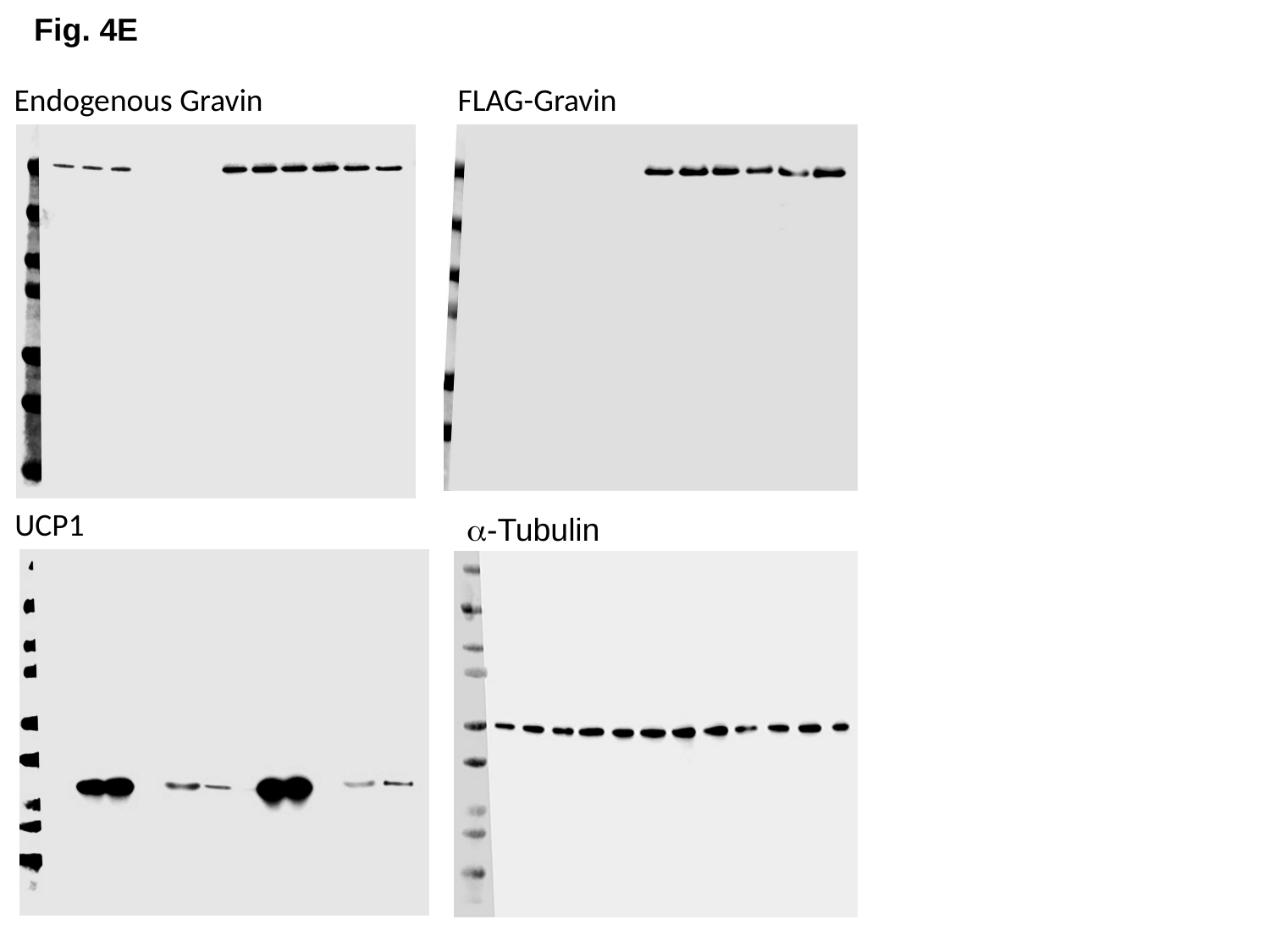

Fig. 4E
Endogenous Gravin
FLAG-Gravin
UCP1
a-Tubulin

## Slide 12
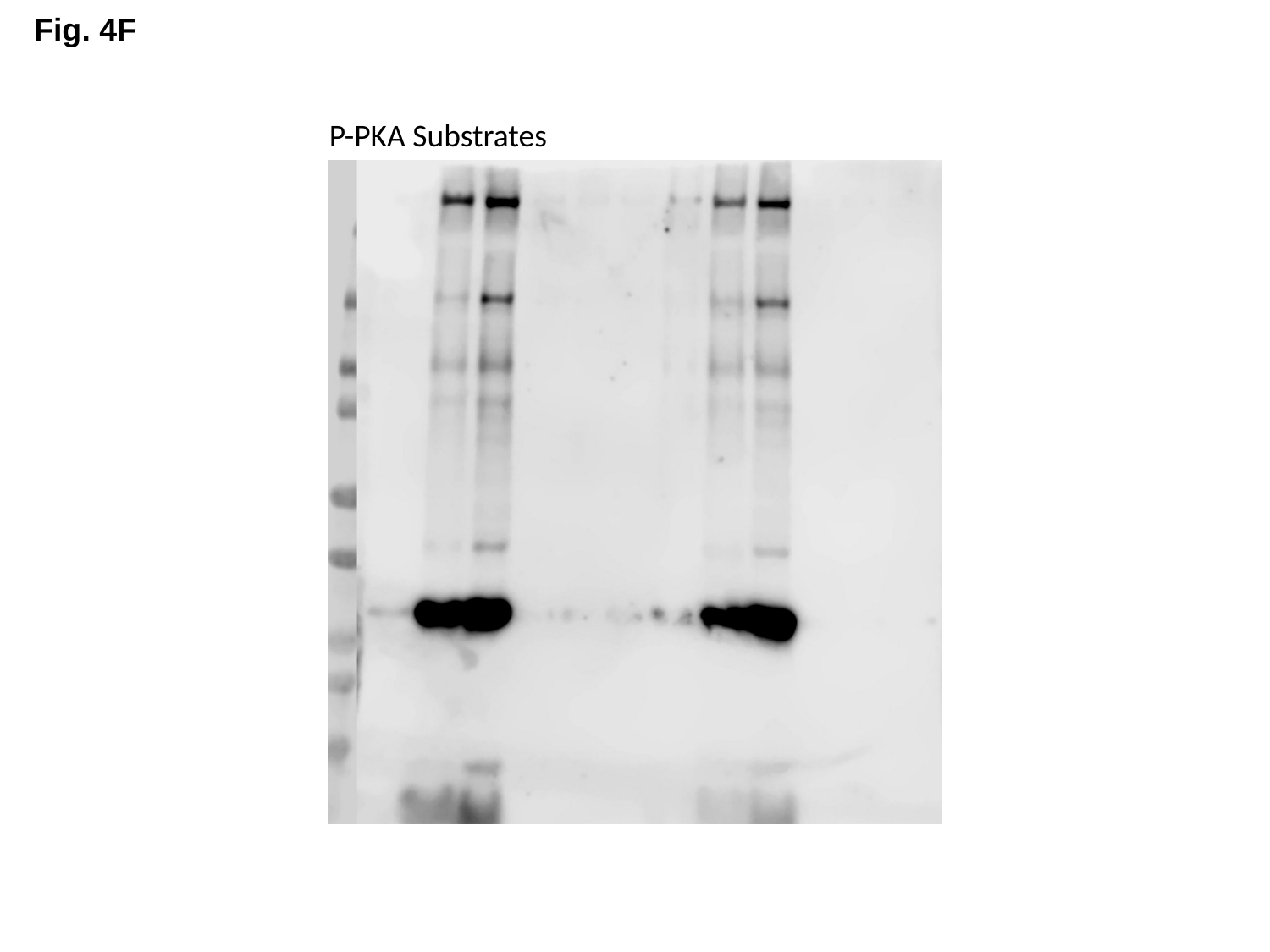

Fig. 4F
P-PKA Substrates

## Slide 13
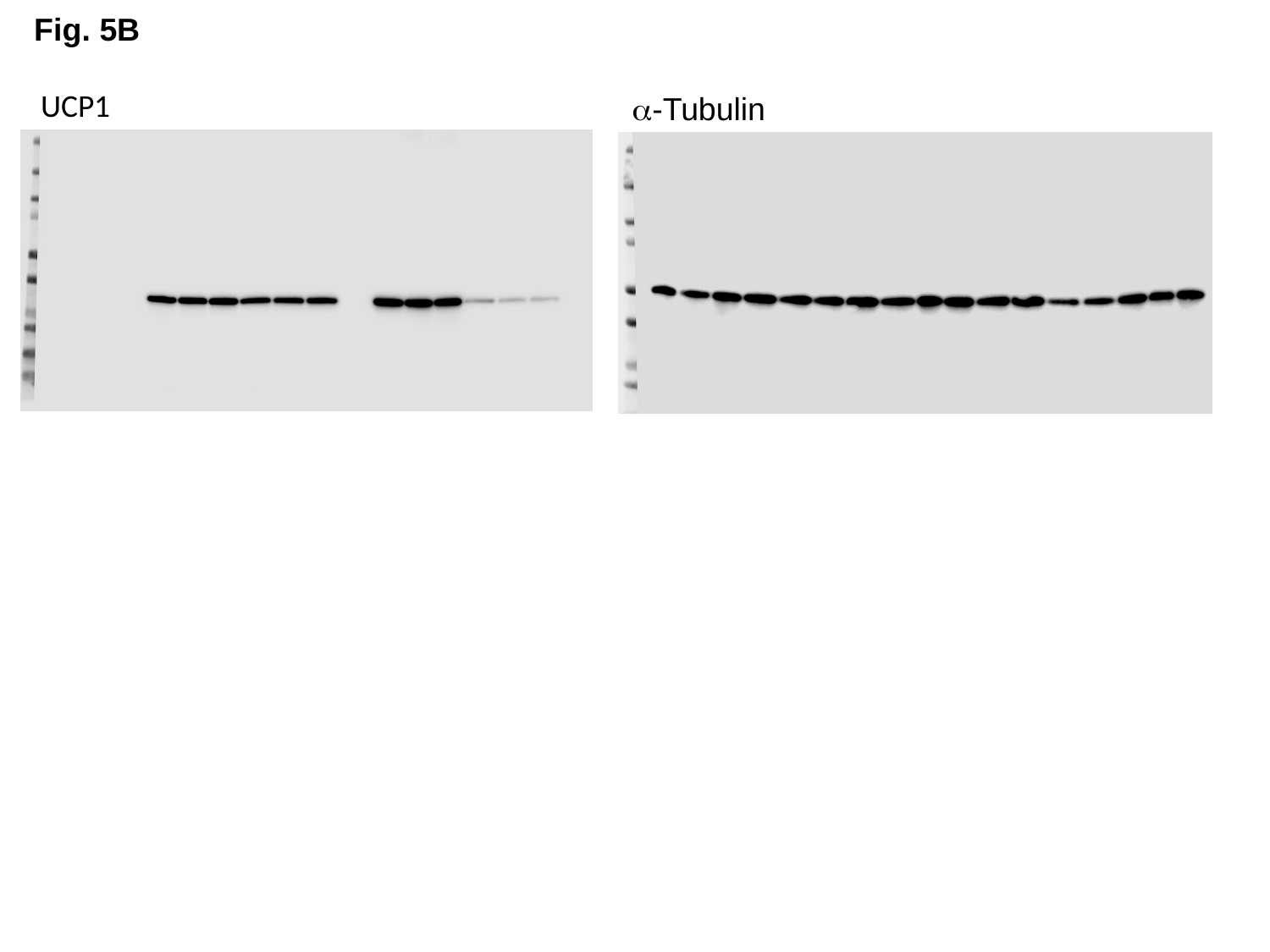

Fig. 5B
UCP1
a-Tubulin

## Slide 14
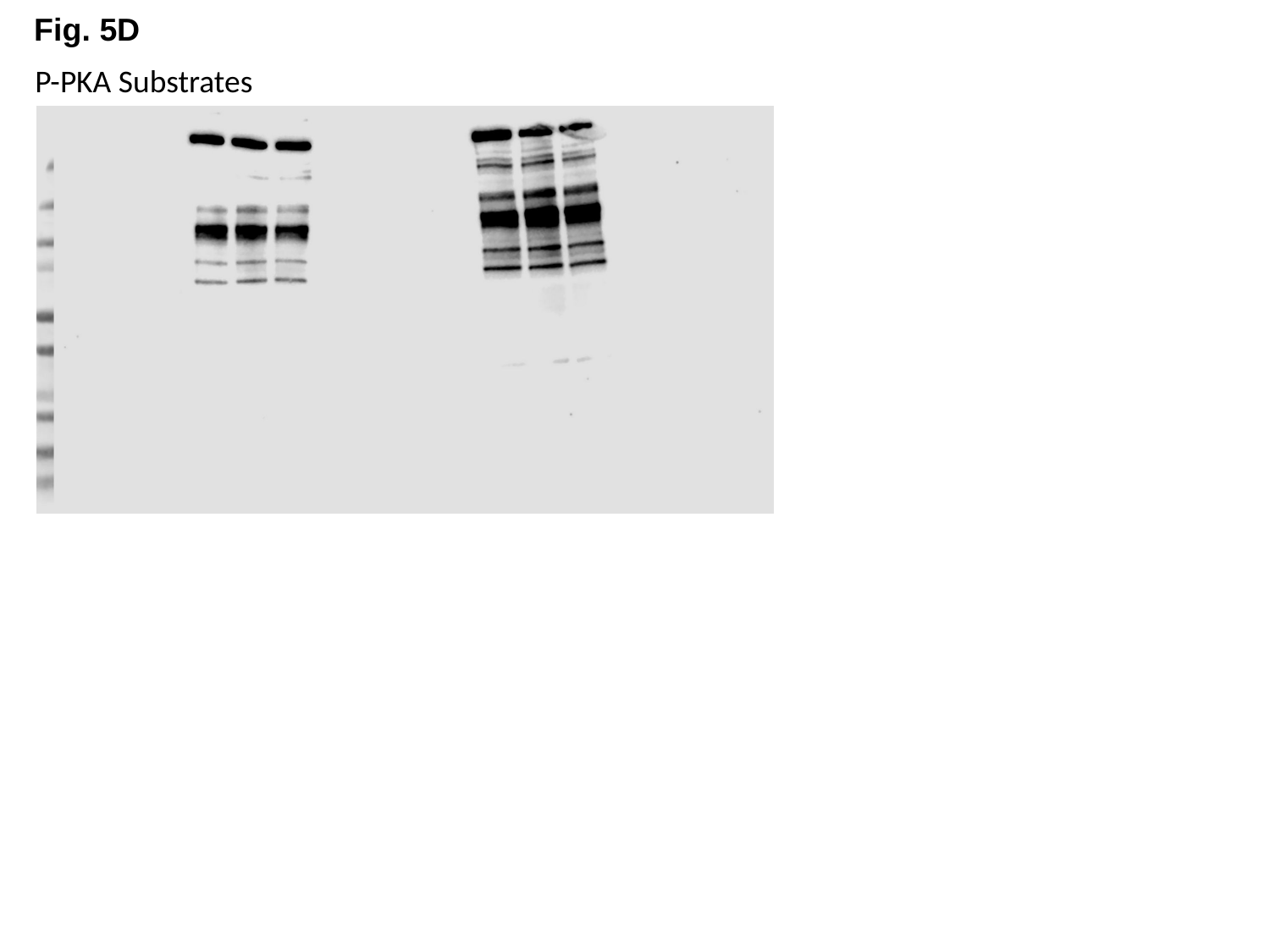

Fig. 5D
P-PKA Substrates

## Slide 15
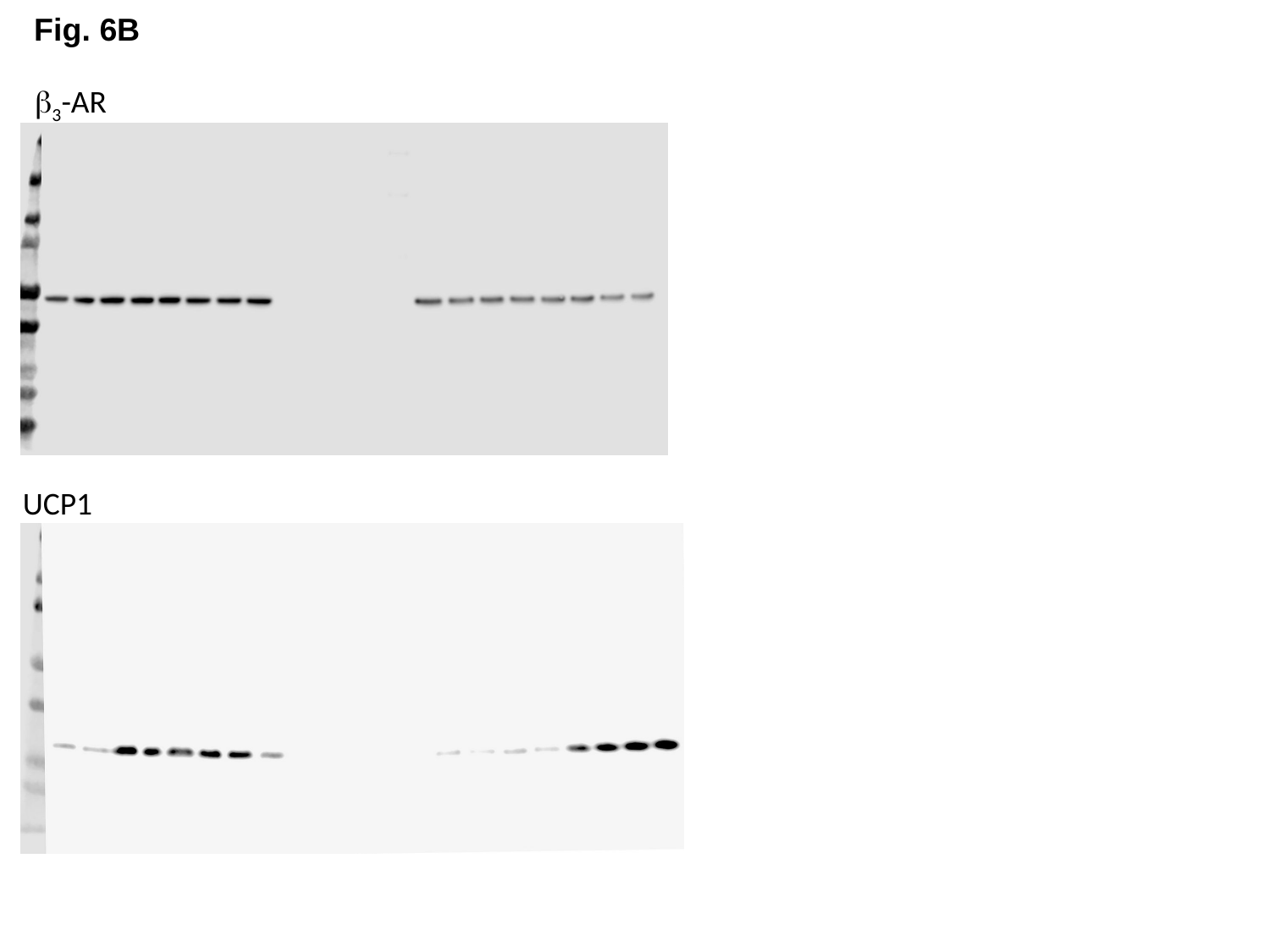

Fig. 6B
b3-AR
UCP1

## Slide 16
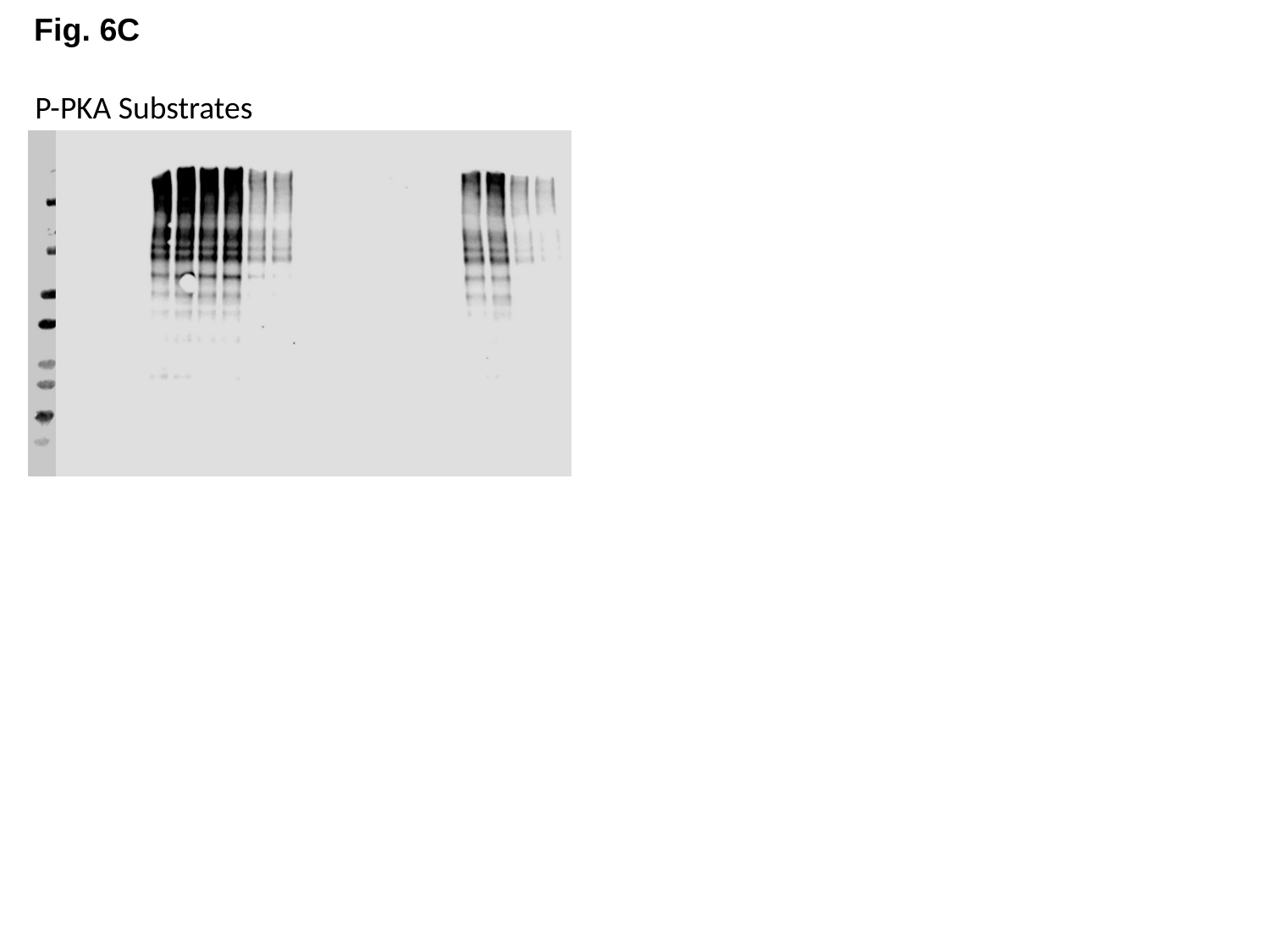

Fig. 6C
P-PKA Substrates

## Slide 17
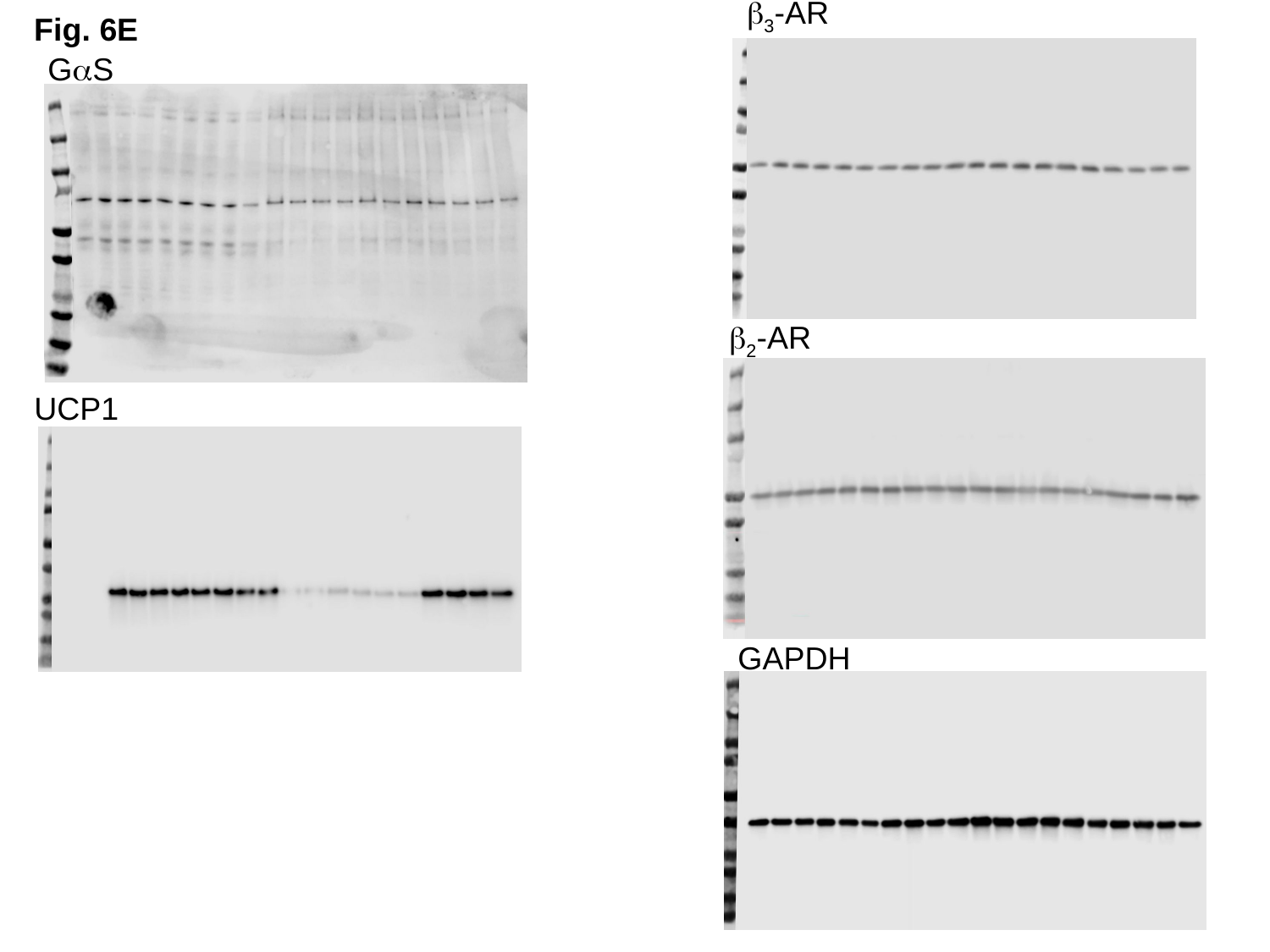

b3-AR
Fig. 6E
GaS
b2-AR
UCP1
GAPDH

## Slide 18
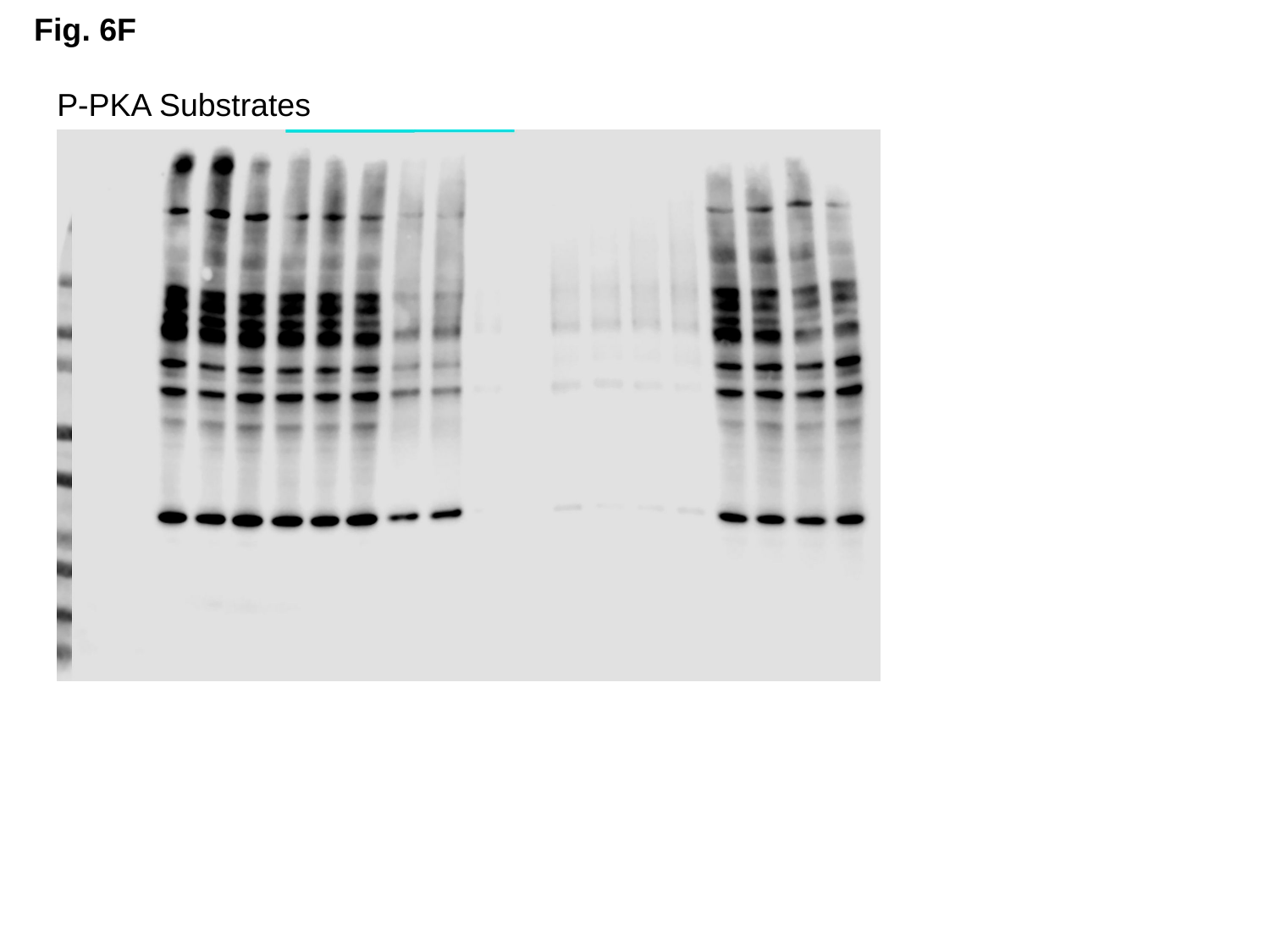

Fig. 6F
P-PKA Substrates

## Slide 19
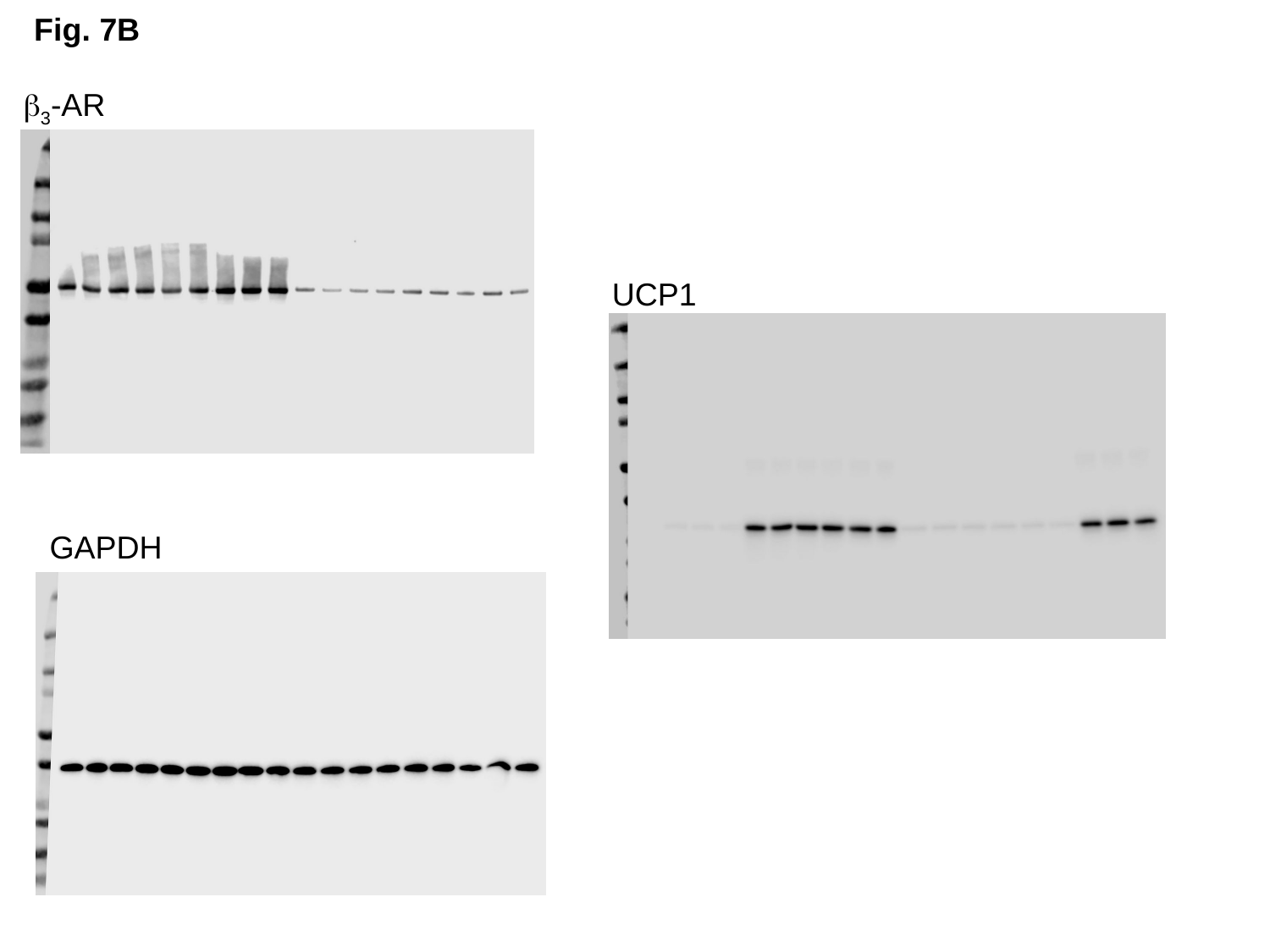

Fig. 7B
b3-AR
UCP1
GAPDH

## Slide 20
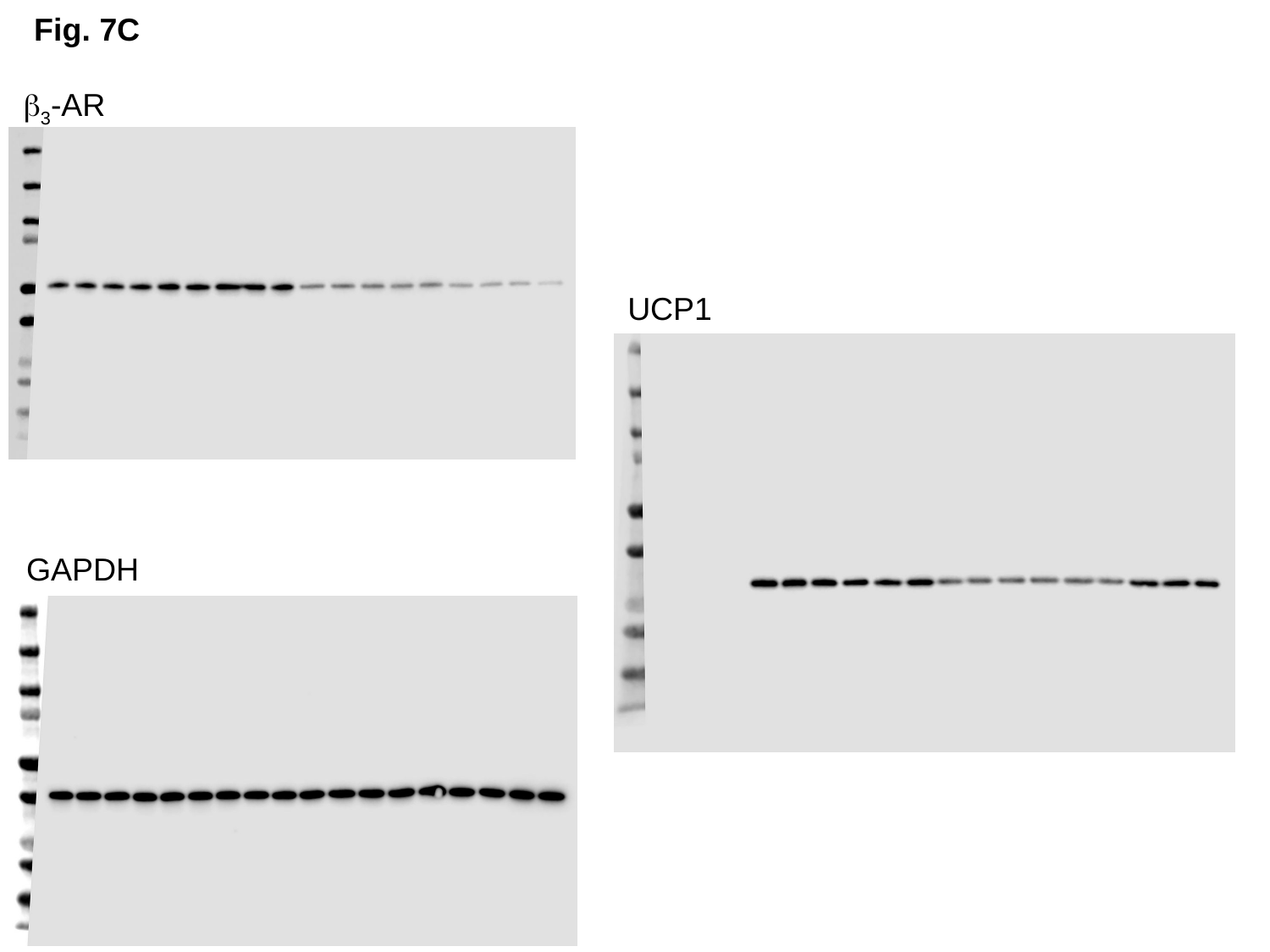

Fig. 7C
b3-AR
UCP1
GAPDH

## Slide 21
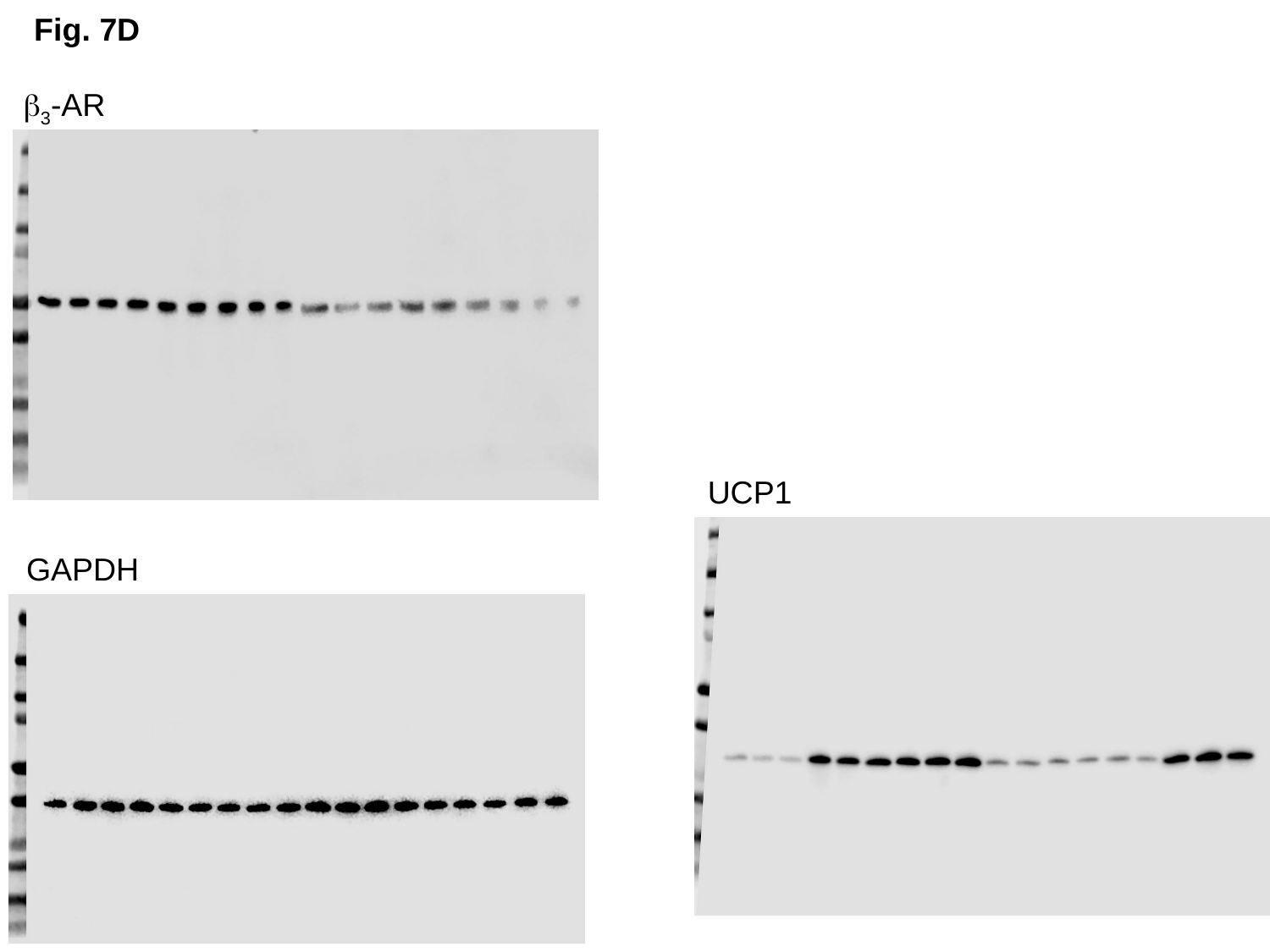

Fig. 7D
b3-AR
UCP1
GAPDH

## Slide 22
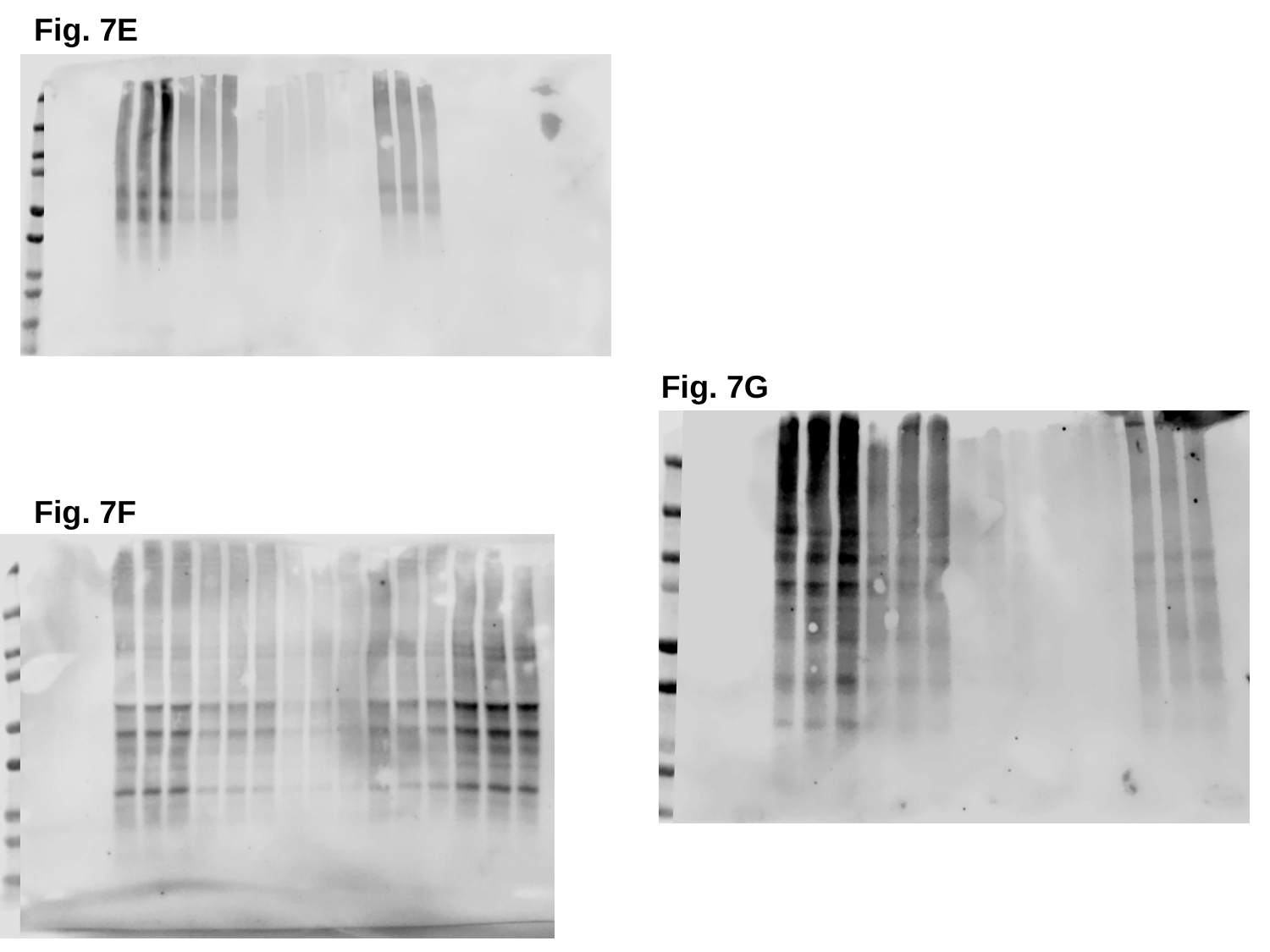

Fig. 7E
Fig. 7G
Fig. 7F

## Slide 23
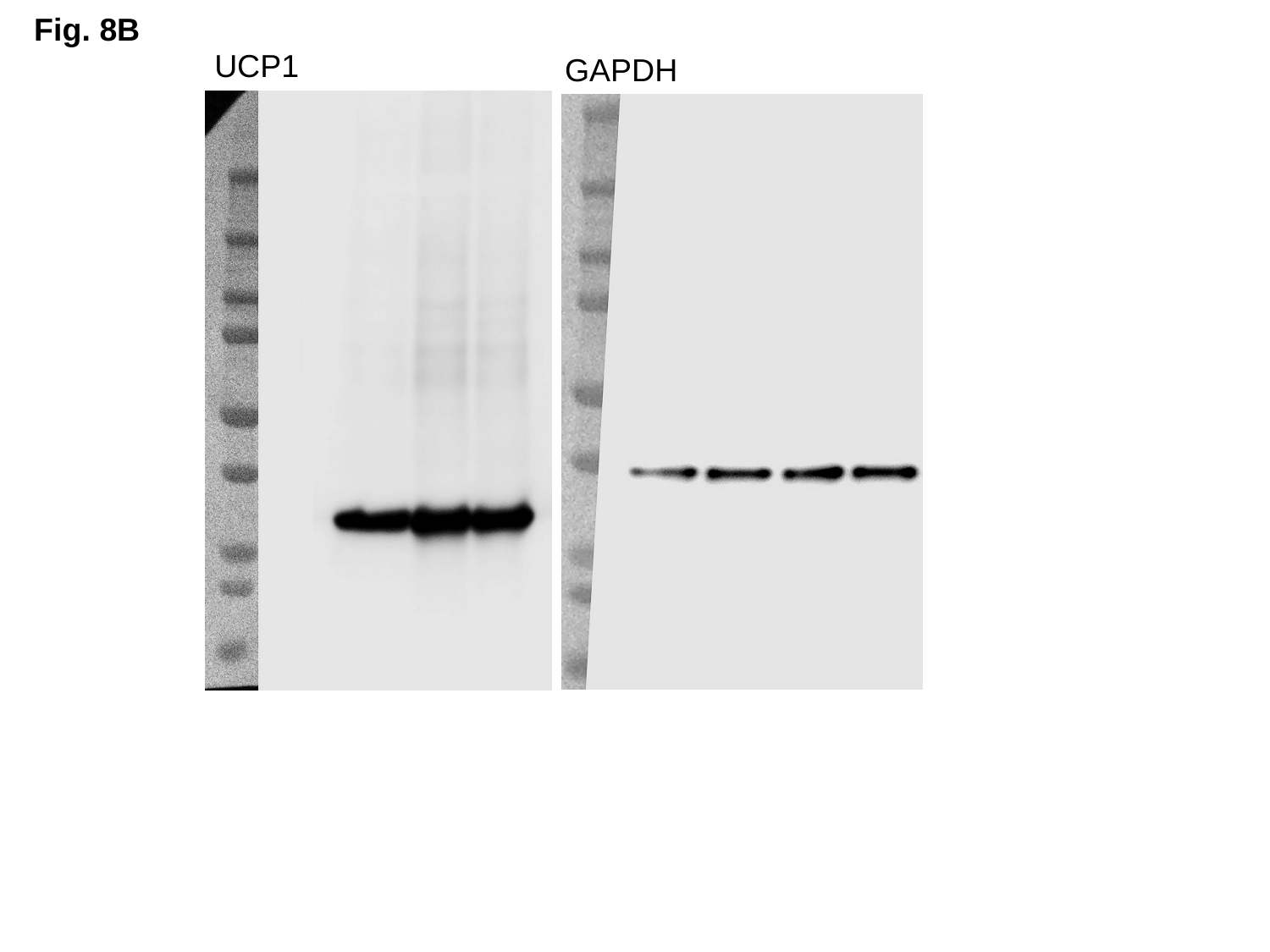

Fig. 8B
UCP1
GAPDH

## Slide 24
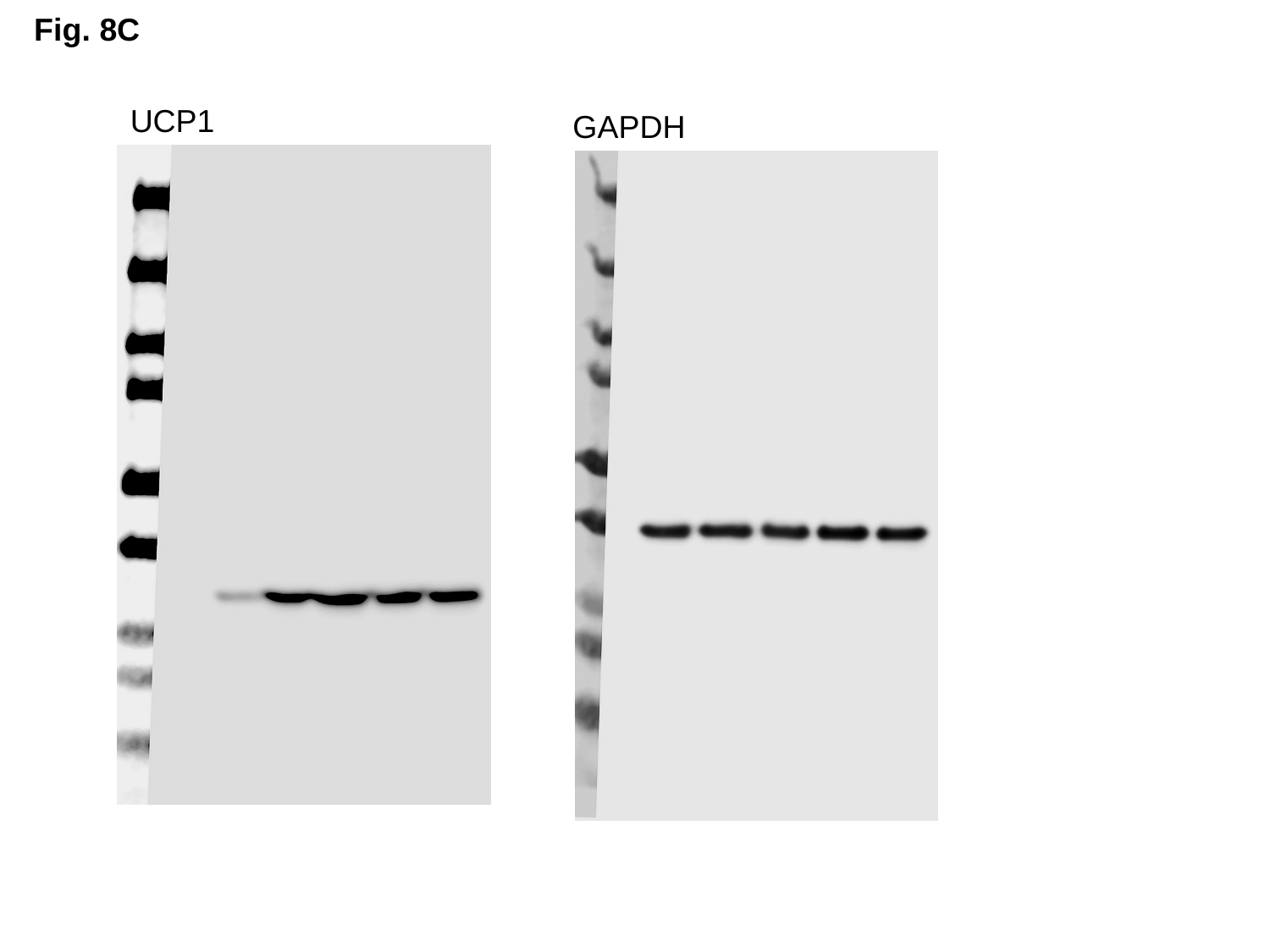

Fig. 8C
UCP1
GAPDH

## Slide 25
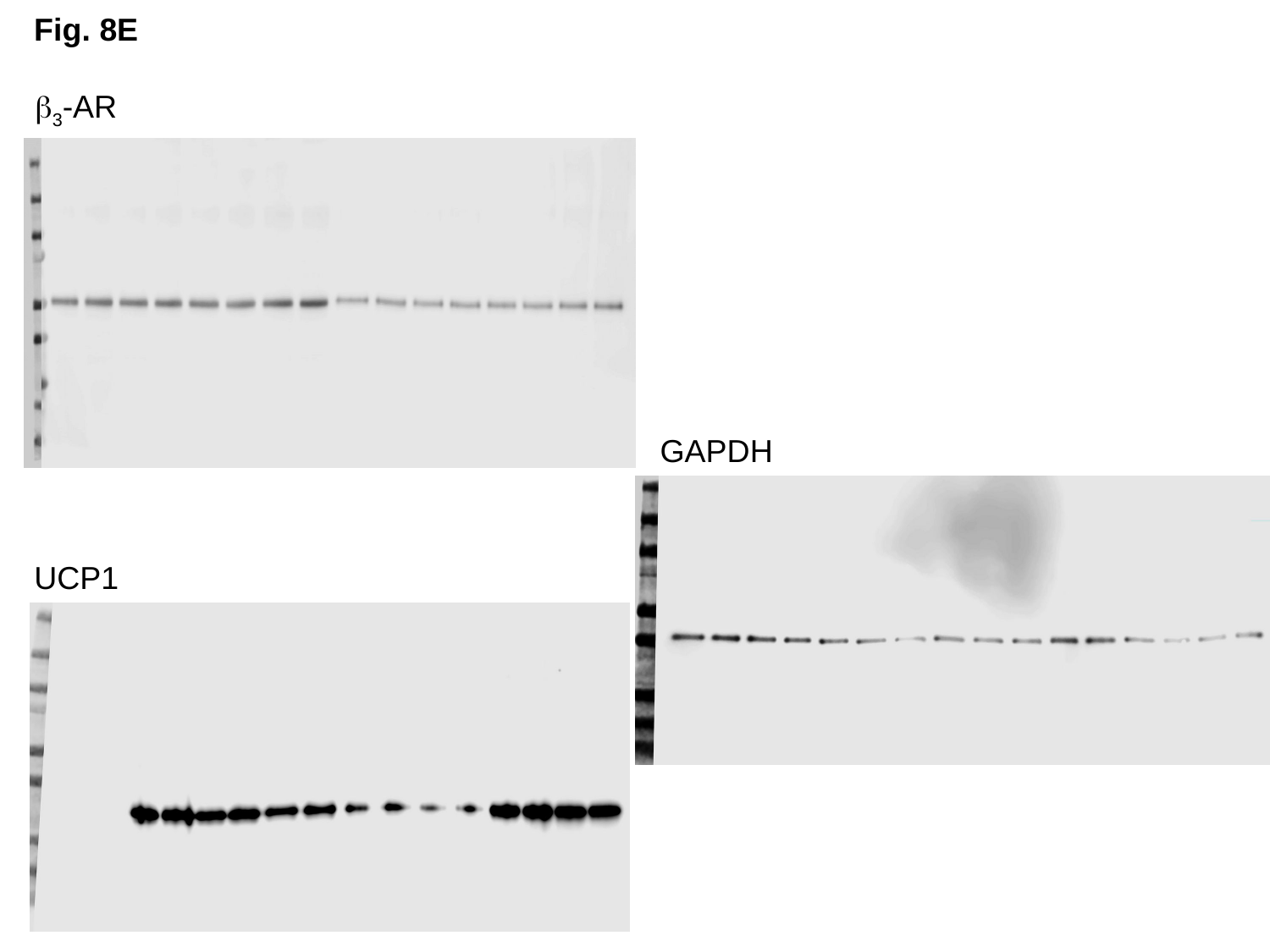

Fig. 8E
b3-AR
GAPDH
UCP1

## Slide 26
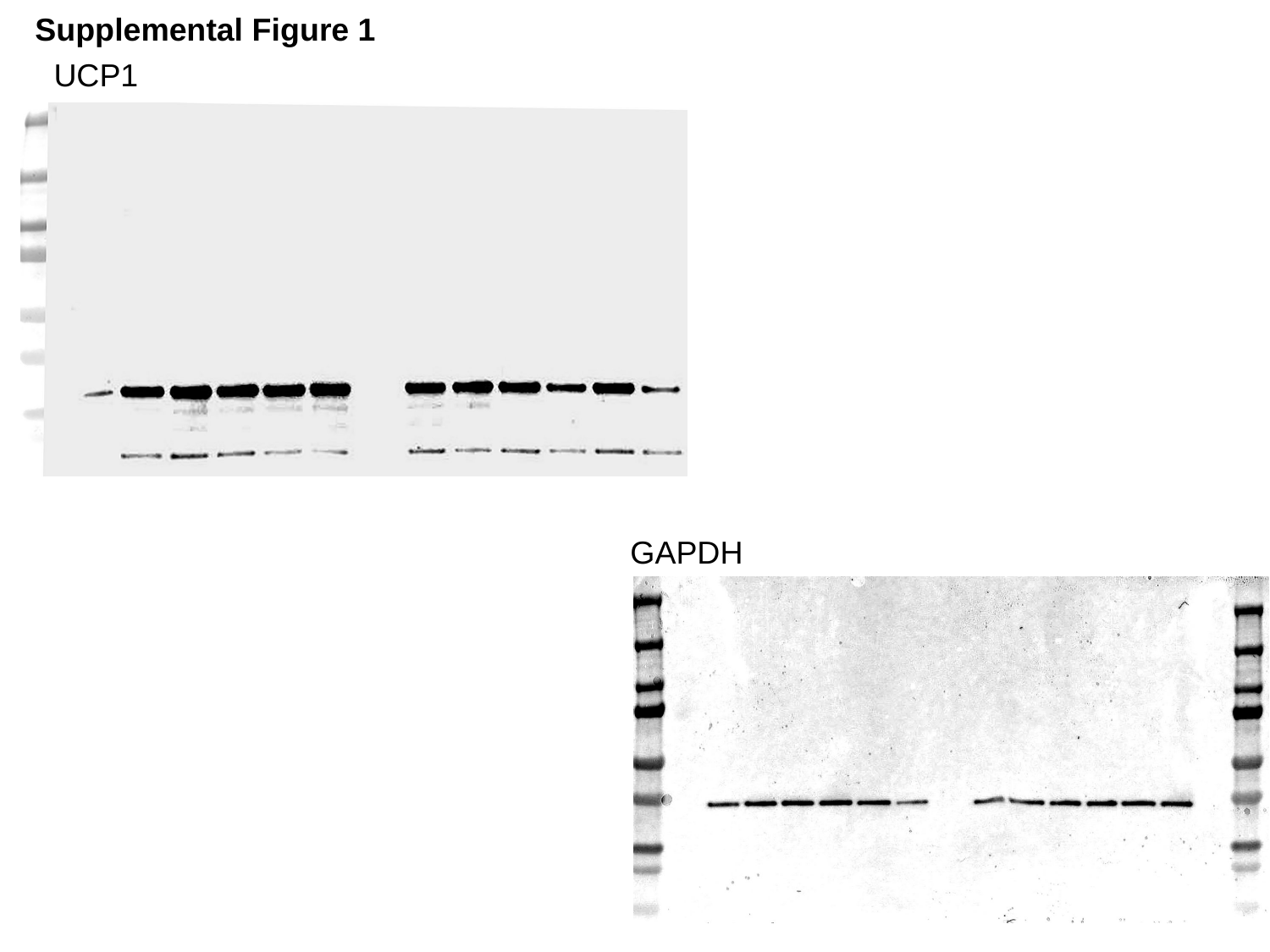

Supplemental Figure 1
UCP1
GAPDH

## Slide 27
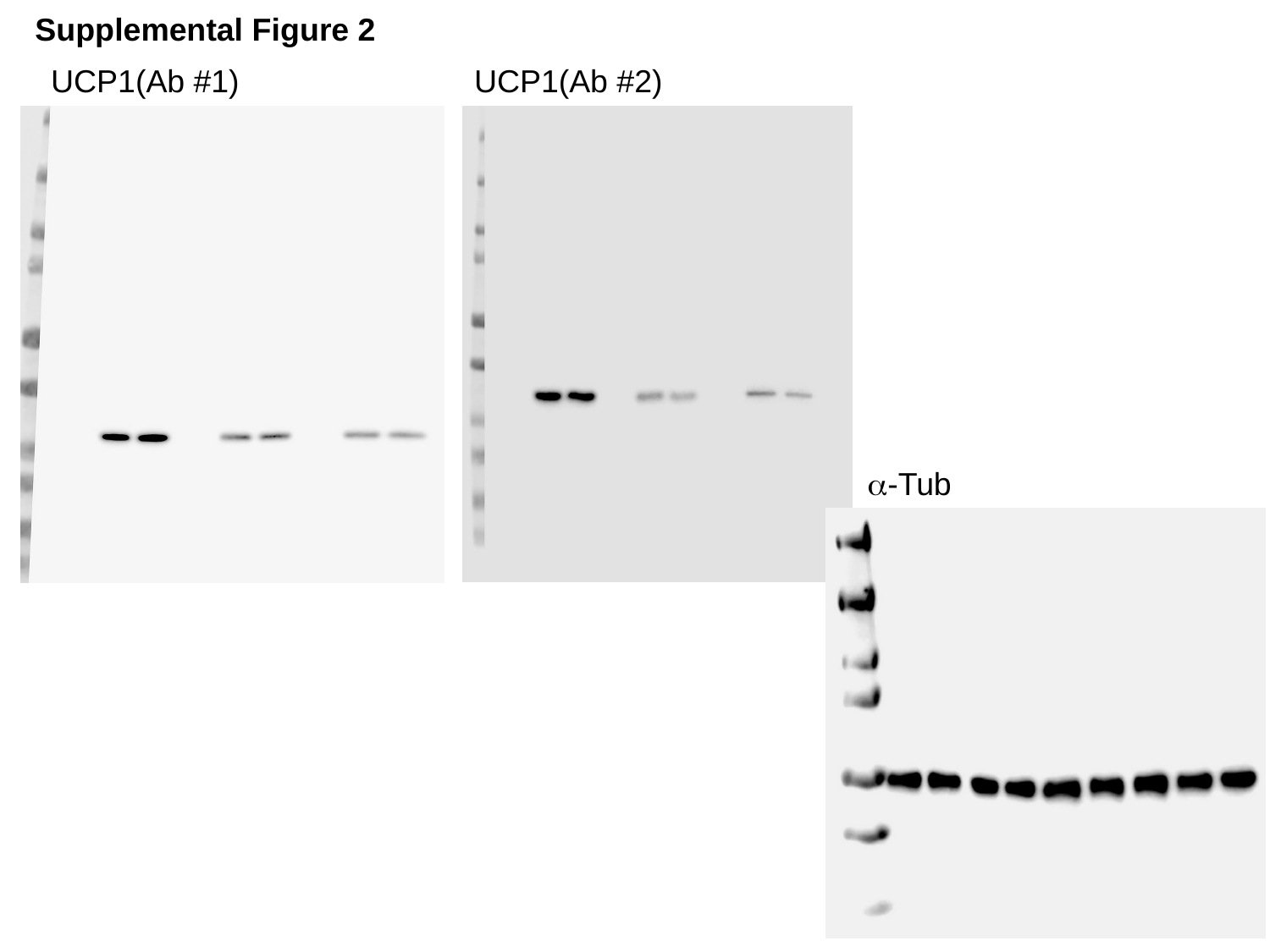

Supplemental Figure 2
UCP1(Ab #1)
UCP1(Ab #2)
a-Tub
